# Mass spectrometry-based proteomic techniques to identify cerebrospinal fluid biomarkers for diagnosing suspected central nervous system infections. A systematic review

**DOI:** 10.1016/j.jinf.2019.08.005

**Published:** 2019-11

**Authors:** Tehmina Bharucha, Bevin Gangadharan, Abhinav Kumar, Xavier de Lamballerie, Paul N. Newton, Markus Winterberg, Audrey Dubot-Pérès, Nicole Zitzmann

**Affiliations:** a Institute of Glycobiology, Department of Biochemistry, South Parks Road, Oxford OX1 3RQ, United Kingdom; b Lao-Oxford-Mahosot Hospital-Wellcome Trust Research Unit (LOMWRU), Microbiology Laboratory, Mahosot Hospital, Vientiane, Lao Democratic People's Republic; c Unité des Virus Émergents (UVE: Aix-Marseille Univ – IRD 190 – Inserm 1207 – IHU Méditerranée Infection), Marseille, France; d Centre for Tropical Medicine and Global Health, Nuffield Department of Clinical Medicine, University of Oxford, Churchill Hospital, Oxford, United Kingdom; e Mahidol Oxford Tropical Medicine Research Unit, Faculty of Tropical Medicine, Mahidol University, 3/F, 60th Anniversary Chalermprakiat Building, 420/6 Rajvithi Road, Bangkok 10400, Thailand

**Keywords:** Neurological infections, Diagnosis, Mass spectrometry, Biomarkers, Proteomics

## Abstract

**Objectives:**

Central nervous system (CNS) infections account for considerable death and disability every year. An urgent research priority is scaling up diagnostic capacity, and introduction of point-of-care tests. We set out to assess current evidence for the application of mass spectrometry (MS) peptide sequencing in identification of diagnostic biomarkers for CNS infections.

**Methods:**

We performed a systematic review (PROSPERO—CRD42018104257) using PRISMA guidelines on use of MS to identify cerebrospinal fluid (CSF) biomarkers for diagnosing CNS infections. We searched PubMed, Embase, Web of Science, and Cochrane for articles published from 1 January 2000 to 1 February 2019, and contacted experts. Inclusion criteria involved primary research except case reports, on the diagnosis of infectious diseases except HIV, applying MS to human CSF samples, and English language.

**Results:**

4,620 papers were identified, of which 11 were included, largely confined to pre-clinical biomarker discovery, and eight (73%) published in the last five years. 6 studies performed further work termed verification or validation. In 2 of these studies, it was possible to extract data on sensitivity and specificity of the biomarkers detected by ELISA, ranging from 89–94% and 58–92% respectively.

**Conclusions:**

The findings demonstrate feasibility and potential of the methods in a variety of infectious diseases, but emphasise the need for strong interdisciplinary collaborations to ensure appropriate study design and biomarker validation.

## Highlights

• We performed a systematic review of MS-based peptide sequencing to identify cerebrospinal fluid biomarkers to diagnose neurological infections.
• Eleven studies were identified, with results demonstrating feasibility and potential for diagnosis of a range of aetiologies, including tuberculosis, West Nile virus infection and trypanosomiasis.
• Six studies performed further work termed verification or validation. In two of these studies, it was possible to extract data on sensitivity and specificity of the biomarkers detected by ELISA, ranging from 89–94% and 58–92% respectively.
• We highlight the need for strong interdisciplinary collaboration and investment in these studies, to ensure appropriate study design, and see biomarkers progress through to validation.
• Successful CSF protein biomarkers could potentially be detected by MALDI-ToF, ELISA and point-of-care immunochromatographic tests.

## Introduction

Central nervous system (CNS) infections account for considerable death and disability every year.^1^ This group of diseases also unduly affects the poorest, marginalised communities, with limited access to healthcare.^2^ An urgent research priority is scaling up diagnostic capacity, particularly in resource-constrained settings, and introduction of point-of-care tests.^2^, ^3^, ^4^, ^5^ Diagnostics are fundamental for individual patient treatment decisions, estimations of disease burden, vaccine effectiveness and treatment trials.

Development and implementation of diagnostics for CNS infections is fraught with difficulties irrespective of the resources available.^6^, ^7^ Brain biopsy is the gold standard for diagnosis, however this is rarely performed.^8^ Clinicians rely on lumbar punctures (LP) to obtain cerebrospinal fluid (CSF) to be tested as a surrogate specimen to test for markers of CNS infection. Furthermore, although currently available methods involve a targeted approach, ‘you need to know what you are looking for’, the list of potential pathogens implicated in CNS infections is extensive, geographically diverse, and dynamic.^9^ The targeted methods largely rely on molecular assays (mainly quantitative real-time polymerase chain reaction, qPCR, assays) or serology (mainly enzyme-linked immunosorbent assays). There are limitations in the accuracy of these tests; broadly, PCR has variable sensitivity in CSF due to low pathogen concentrations, and serology may be poorly specific. Even in the best-resourced centres worldwide, suspected cases of CNS infections remain undiagnosed in one to two-thirds of patients.^7^, ^10^

**Fig. 1:**
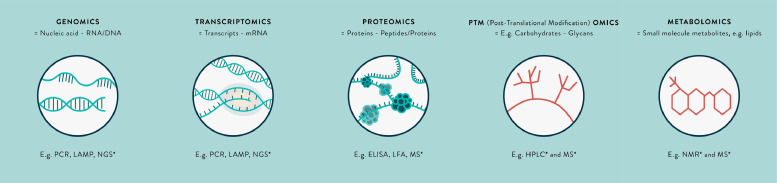
The omics revolution in biomarker discovery – a summary of key terms. *Untargeted-omics techniques. Abbreviations: RNA=Ribonucleic acid; DNA=Deoxyribonucleic acid; mRNA=Messenger RNA; PCR=Polymerase chain reaction; LAMP=Loop-mediated isothermal amplification; NGS=Next-generation sequencing; ELISA=Enzyme-linked immunosorbent assay; LFA=Immunochromatographic lateral flow assay; MS=Mass spectrometry; NMR=Nuclear magnetic resonance.

Metagenomics techniques (Fig. 1), such as next-generation sequencing, are emerging as a novel useful untargeted, ‘you don't need to know what you are looking for’ approach to diagnosing CNS infections.^9^, ^11^, ^12^ In particular, they have a role in identifying pathogens for which there are mutations in the PCR primer or probe binding sites on the genome,^13^ or those that are rare or novel causes and not routinely tested.^14^, ^15^, ^16^, ^17^, ^18^ Currently, evidence from metagenomics is limited to case reports or case series, and testing requires considerable resources and expertise. Furthermore, there are infections (e.g. *Japanese encephalitis virus, JEV*) in which the window for pathogen detection is narrow, and a diagnostic test based on identifying genetic material has limited use. Advances in molecular technology are likely to reduce the proportion of undiagnosed CNS infections, however it is likely that at least 10–20% will remain undiagnosed by these methods.^9^

Alternative methodological approaches involve transcriptomics, proteomics and metabolomic techniques (see Fig. 1).^11^, ^19^ These may identify pathogen-derived, or host markers of infection as mRNA transcripts, proteins or metabolites, respectively, that are altered in expression in certain CNS infections. These biomarkers represent fundamental processes occurring in cells, that is, the functional elements of the genome. The transcriptome is highly dynamic, affected by external perturbations, and mRNA as a biomarker is relatively easily degraded in the extracellular environment.^20^, ^21^ There is minimal literature on the CSF transcriptome. However, studies in whole blood show discrepancies between findings in different studies. Importantly, mRNA detection is not currently as amenable for translation to the bedside as that for a protein biomarker, for which immunochromatographic antibody-based assays ‘pregnancy test style dipsticks’ may be produced. There are similar issues with metabolomics, the study of substrates and products of metabolism, and sample preparation is more complex.^19^, ^22^ For these reasons, this review is focussed on proteomics, although the potential of transcriptomics and metabolomics in this field remains to be explored. An illustration of the potential impact of a protein biomarker is the detection of non-structural protein 1 (NS1) in suspected dengue virus infection. The window for detection of NS1 protein is longer than for dengue virus RNA, and the protein has been easily harnessed for point-of-care testing.^23^ NS1 protein ELISA and rapid tests have been introduced worldwide for testing serum and CSF, as recommended by the World Health Organisation.^24^ Similar successes for other pathogens could have significant incremental public health impact.

Analysis of CSF total protein concentration is one of the cornerstones of conventional diagnostic techniques, used to classify CNS infections into bacterial, tuberculous and viral categories.^25^, ^26^ However, beyond serological testing, to date there has been limited unbiased profiling of the CSF proteome in infections.^27^ One of the reasons for the lack of knowledge in the field of protein biomarker development has been limitations of available technology, including challenges in substantial variability in concentration of different proteins in clinical samples and low concentration of potential biomarkers, i.e. ‘trying to find the needle in a haystack’. Advances in mass-spectrometry (MS) instruments and workflows has transformed proteomics applications, which means that we are now in a position to start to address these issues. MS proteomics techniques facilitate uniquely unbiased, sensitive and quantitative analysis of body fluids and tissues. This is evident from the enormous investment in proteomics biomarker discovery for neurodegenerative diseases and cancer.^28^

**Fig. 2:**
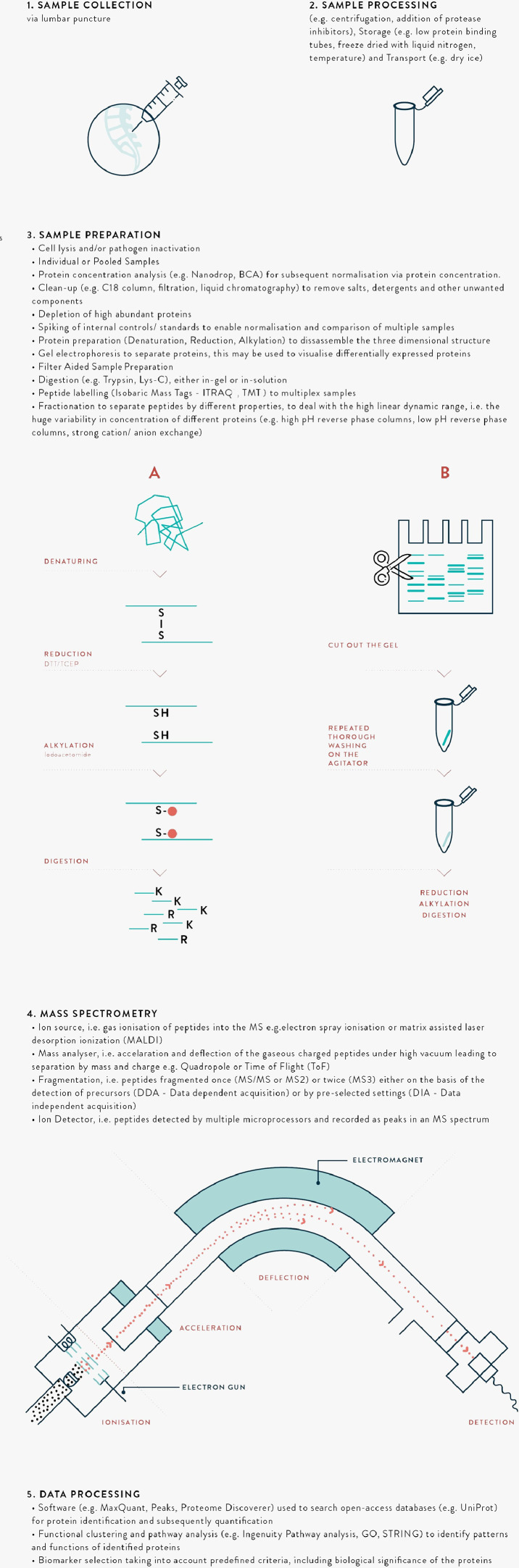
Schematic diagram of the key steps in mass spectrometry CSF biomarker identification.

There are a wide range of MS proteomics methods involving ‘top-down’, intact protein analysis, or ‘bottom-up’ (protein digestion and sequencing) approaches. A glossary of MS terminology is presented in Table 1, with schematic illustration of the processes in Fig. 2. The application of Matrix-Assisted Laser Desorption/Ionization-Time Of Flight MS (MALDI-TOF) has revolutionised the medical microbiology laboratory due to the speed, accuracy and low cost of processing, although a limitation is that it is currently used in clinical practice only for pathogens included in the database.^29^ For biomarker discovery, the principal approach incorporates bottom-up MS, typically coupled with nanoflow liquid chromatography to separate samples into fractions and address the substantial differences in abundances of different proteins, referred to as the high dynamic range.^30^ An important technique has been the introduction of peptide labelling, allowing introduction of multiple samples each with different labels during a single mass spectrometry run, improving comparison of relative protein abundance between samples. There have also been considerable advances in separation methods to enable deeper probing of the proteome.^31^

**Table 1:** Glossary of MS terminology.

Term	Explanation
Biomarker	A characteristic that is used as an indicator of normal biological processes, pathogenic processes or responses to a therapeutic intervention.
Mass spectrometry (MS)	The basic principle underlying MS is the identification of peptides or proteins based on separation by mass and charge. Peptides or proteins are ionised, accelerated and usually deflected by a strong electromagnetic field, such that they reach a detector at different times based on their mass and charge. Detection of a peptide or protein is recorded as a peak, and used to determine the peptide sequence and identity. This may also be termed peptide mass fingerprinting, or peptide sequencing.
Types of Ionisation; ESI, MALDI and SELDI.	Introduction into the MS of sample containing proteins or peptides of interest requires ionisation. Two key methods include 1. electron spray ionisation 'ESI' and 2. laser desorption ionisation (matrix-associated ‘MALDI’ and surface-enhanced ‘SELDI’). MS involves different combinations of ionisation and mass analysis, see below.
Tandem MS; MS2 and MS3	MS may also involve the fragmentation of peptide or protein ions, such that a precursor peak is recorded along with fragment peaks (MS2) or fragments of fragments (MS3). This improves selectivity, identification and detection.
Types of Mass Analysers; Quadrupole, ToF and Orbitrap	Mass analysers separate peptide or protein ions within the MS. Two key methods include 1. quadrupole, four metal rods with alternating electromagnetic fields, and 2. time of flight, based on time alone. 3. Orbitrap, based on ionised ions rotate around a central rod at high voltage and vacuum. MS may involve a single or combination of methods.
Top-down vs. Bottom-up	Prior to MS, samples may be digested with an enzyme (usually trypsin) into peptide fragments. This is termed bottom-up MS. In contrast, if there is analysis of intact proteins or it is termed top-down MS. Bottom-up is more common, however there are instances when top-down is required, for example for analysis of proteins in their native structure.
Unbiased vs Targeted (e.g. PRM, SRM, MRM)	MS performed to identify specific pre-selected proteins or peptides is termed targeted, whereas discovery MS performed to identify any/ novel proteins or peptides is termed unbiased or shotgun 'you don't need to know what you are looking for'. Targeted MS is usually more sensitive than unbiased, and also allows high-throughput, e.g. parallel reaction monitoring (PRM), selected reaction monitoring (SRM) and multiple reaction monitoring (MRM).
Dynamic range	The range in concentrations of proteins present in a sample. This may vary by many orders of magnitude, leading to difficulties in identifying low abundant proteins of interest.
Sample preparation	Steps involved in preparing a sample prior to introduction in the MS. This usually involves disassembly of the 3D structure with denaturation (unfolding; e.g. urea), reduction (breaking disulphide bonds in the secondary structure e.g. dithiothreitol) and alkylation (capping free thiol chains to prevent reformation of disulphide bonds). The protein sheets are then digested into smaller (e.g. 9 amino acid chain) peptide fragments.
Depletion	Removal of high abundant proteins (e.g. albumin and IgG), to improve MS identification of low abundant proteins of interest. Typically involves immunodepletion, i.e. antibody methods.
Pooling	Combining several samples in a single analysis.
Labelling	Addition of isobaric mass tags to peptides during sample preparation, e.g. iTRAQ or TMT, to enable the analysis of multiple samples in a single MS run. This allows relative quantitation of peptides or proteins, reduces the time and obviates bias due to inter-run variation.
Separation and Fractionation; Liquid-chromatography (LC)	In order to deal with the high linear dynamic range in clinical samples, proteins are separated by various methods based on different properties of the proteins such as their mass, charge or hydrophobicity. Multidimensional separation refers to multiple methods being performed on the same sample, and orthogonal when the methods are based on different properties. These methods may be prior to digestion, such as by gel-electrophoresis, after which the gel is cut up and prepared to be loaded on the MS. There are various methods coupled with the mass spectrometry machine, e.g. liquid chromatography. e.g. strong cation exchange, high pH or low pH reverse-phase and C18 analytical column.
Functional analysis or Clustering	Identified proteins are investigated using programs that analyse the subcellular location, biochemical pathways and functions. Clustering refers to the mapping of proteins by their reported functions, to examine whether multiple proteins are involved in similar pathways, as this may strengthen evidence for their roles.

**Fig. 3:**
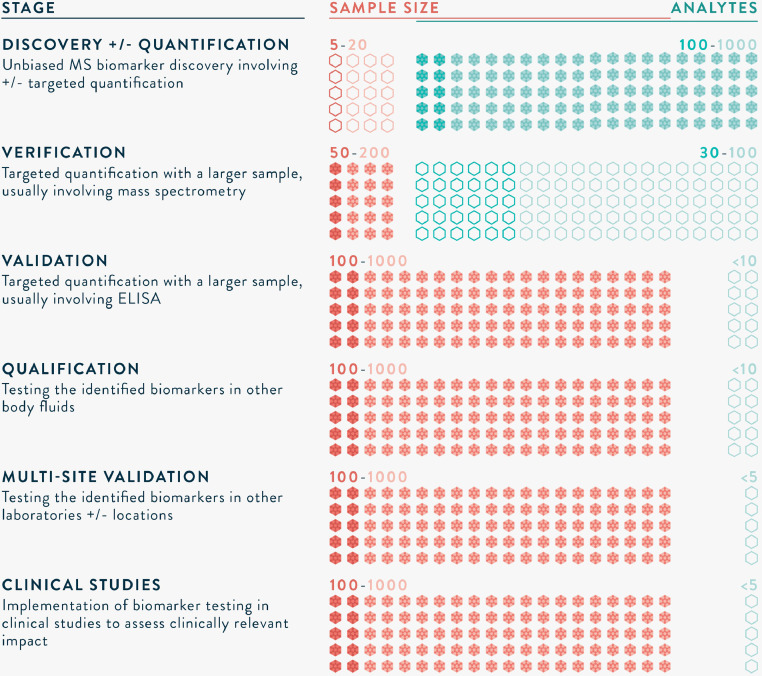
The long road to clinical application of biomarkers.^32^ *Sample sizes are informed by appropriate statistical tests, and numbers provided are rough estimations of those required.

MS-based proteomics biomarker development has aptly been compared to the drug development pipeline. Illustrated in Fig. 3, a series of well-designed studies are needed, from biomarker discovery through to implementation in clinical practice.^32^ Appropriate sample size calculations are required to ensure that inferences are valid. A discovery study typically incorporates a small number (e.g. 5–20) of samples (carefully selected cases and controls) and unbiased, highly sensitive MS workflows. Candidate biomarker(s) identified are then verified in a larger group (e.g. 50–200 samples) using high-throughput techniques such as parallel reaction monitoring (PRM). Machine learning algorithms, such as principal component analysis, are frequently applied to identify biomarker(s) that best differentiate between cases and controls.^33^ Based on calculations of receiver operating characteristics (ROC) curves, with targets for sensitivity and specificity, a biomarker or a combination of biomarkers are selected. The proteins are usually utilised to develop an antibody-based test, such as an ELISA or immunochromatographic assay. Validation involves testing in an even larger group (e.g. 100–1000 samples), in multiple sites, and may also be qualified in other body fluids. Prior to implementation in clinical practice, well-designed studies are also needed to demonstrate clinical impact and/or cost-effectiveness. As with drug-development, many candidate biomarkers do not reach clinical practice, for a variety of reasons such as lack of sensitivity, specificity or reproducibility, however this is an inevitable part of the process.

We performed a systematic review evaluating the use of mass spectrometry-based proteomics techniques to identify biomarkers in CSF for the diagnosis of suspected CNS infections. The review covered all ages, and included clinical studies from biomarker discovery through to validation. The aim was to summarise the state of this promising technology, to identify problems and focus on research needed to improve its implementation into clinical practice.

## Methods

The study protocol was registered with PROSPERO, study ID CRD42018104257. References for this review were identified through searches of PubMed, Embase, Web of Science, and Cochrane for articles published from 1st January 2000 to 1st February 2019, by use of the Medical Subject Headings (MeSH) and text word terms (“cerebrospinal fluid” or “csf”), (“mass-spectrometry” or “proteom*” or “biomarker*”) and “infectio*”. Criteria for study inclusion involved all primary research except case reports, on the diagnosis of infectious diseases except HIV, applying mass-spectrometry to human cerebrospinal fluid samples and reporting in English language. Articles were imported into EndNote, and then de-duplicated. Abstracts were screened for the study criteria. Two authors (TB and BG) independently performed a review of full articles to identify eligible articles using the study criteria, and then used standardised proformas to perform quality assessment and data extraction. A third author (AK) resolved any disagreement. Additional articles were identified from hand-searching references in relevant articles, searching Web of Science for publications citing the articles, and by emailing experts in the field. Quality assessment was performed in accordance with the guidelines, ‘Recommendations for Biomarker Identification and Qualification in Clinical Proteomics’^34^ using the standardised eight-point checklist. Another reporting guideline has also been utilised in the discussion, the ‘Guidelines for uniform reporting of body fluid biomarker studies in neurologic disorders’,^35^ and relates to mature biomarker candidates.^36^ Data were extracted using criteria developed by the authors in reference to available guidelines.^32^, ^34^, ^35^^,^^37^, ^38^, ^39^ During the data extraction, authors of relevant articles were contacted for further details, if required.

## Results

### Study selection

**Fig. 4:**
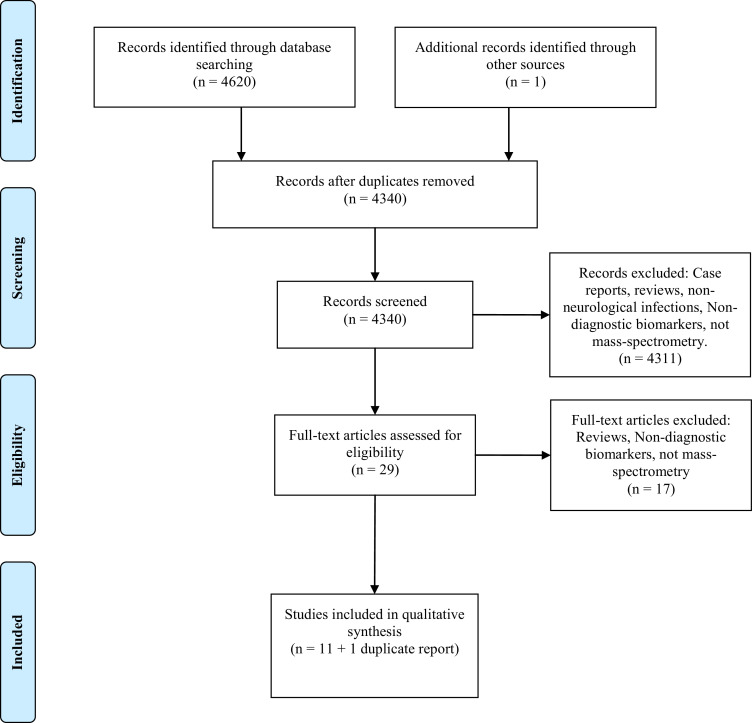
PRISMA flow diagram of the number of records identified, included and excluded, and the reasons for exclusions.

A PRISMA Flow diagram is presented in Fig. 4, and summarises the results at each stage in the process of study selection. Database searches identified 4,620 papers (PubMed=1,086, Embase=2,885, Web of Science=649, Cochrane=0). De-duplicating and initial screening of abstracts as per the inclusion criteria identified 29 papers. The majority of the identified papers were excluded during screening as they did not involve primary research, neurological infections, or mass-spectrometry. For instance, many focused on cell culture proteomics or multiplex ELISA techniques. Full-text screening was performed using the same inclusion criteria, and led to the inclusion of 12 papers for quality assessment and data extraction.^40^, ^41^, ^42^, ^43^, ^44^, ^45^, ^46^, ^47^, ^48^, ^49^, ^50^, ^51^ Articles excluded during the full-text analysis were reviews, involved ELISA or protein microarray techniques, or not confined to diagnostic biomarkers. Two studies^45^, ^51^ reported the same patients and mass-spectrometry experiment, with focus on different findings obtained and different confirmatory methods. The two articles are counted as one study for the purposes of calculated percentages below.

### Quality assessment

**Fig. 5:**
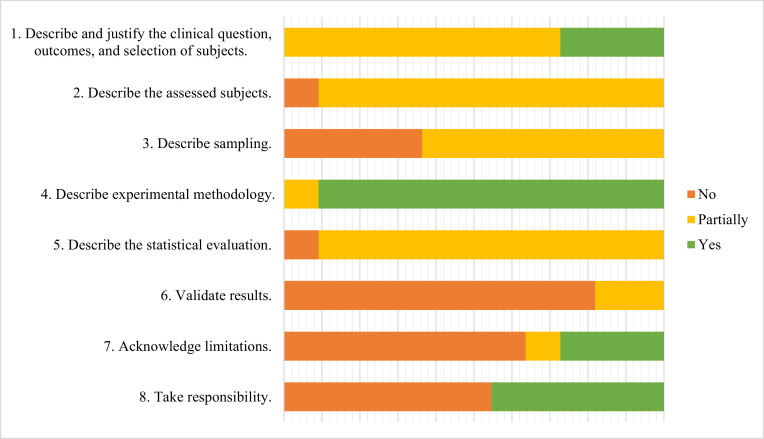
Quality assessment of included studies.

Quality of studies was assessed using the eight criteria set out by the guidelines of Mischak et al., 2010^34^ (Fig. 5, Supplementary Data A). None of the studies fulfilled all the criteria. Eight studies (73%) did not report clear questions, outcomes, or selection of subjects. One study did not report patient age, one study reported the age range, and four studies reported mean patients ages while it is more appropriate to report median ages given the small sample size. Gender was reported, and these were broadly matched, however there was no discussion on this potential effect. Ethnicity was not reported in three quarters of articles. None reported comorbidities, pregnancy or routine medications.

There were minimal data on the methods of sample collection or duration of sample storage. However, the studies provided data on sample storage, preparation, and the technical aspects of mass-spectrometry. None of the studies reported sample size calculations, and inconsistent reporting of statistical methods were used. Although the studies uniformly reported the potential role of identified proteins/peptides, study limitations were only reported in one third of studies.

A fixed quality cut-off for including studies was not set, and all the studies were included in the data extraction. Nonetheless, it is notable that eight (73%) studies failed 6 of 8 points of the quality assessment. This may partly be due to insufficient awareness or adherence to reporting guidelines. However, it also suggests a high-risk of bias across the studies.

### Data extraction

The data extracted from the studies are summarised in Table 2, with further detail in Supplementary Data B. All the identified studies involved biomarker discovery work, and six studies also performed further work termed verification or validation. The studies were published between 2011 and 2018 and eight (73%) were from the last five years. There was a wide geographical spread of locations of mass-spectrometry experiments; five performed in Asia, four performed in Europe, one in North America, one in South America and one in Africa. Most of the samples were collected in the same country in which mass-spectrometry experiments were performed, except for three in which samples were transported from Angola to France, Greece, and Netherlands to France,^43^ from Malawi to the UK,^44^ and from Angola, Chad, and the Democratic Republic of the Congo to Switzerland.^52^ It was not possible to perform a meta-analysis of included studies due to incomplete reporting, and lack of response (2, 18% responded) to email requests sent to corresponding authors.

**Table 2:** Summary of Included Studies

Reference	Site^a^	Study Groups	Sample Size^b^	Methods Summary	Body Fluid	MS	Data Processing		Biomarkers	
							Peptide Search Database	Software	Criteria	Results
Angel 2012	USA	Early-disseminated Lyme disease vs. CSF inflammation	26 vs. 19	Biomarker Discovery:1) CSF from 9 pooled cases vs. 12 pooled controls, fractionated using offline strong cation exchange (SCX) into 35 vs. 25 fractions and analysed label-free by reverse-phase capillary LC-MS; 2) To enable analysis of a larger group including samples with insufficient volume for fractionation, CSF from 26 cases (including the same as the first group) and 7 controls were analysed individually by unfractionated label-free LC-MS.	CSF	LTQ	Human and Pathogen	Identification with SEQUEST,quantification & statistical analysis with in-house DAnTE.	ANOVA (P value <0.05); AUROC >=0.8.	13 host proteins differentially expressed between cases vs. controls: Secreted phosphoprotein 1, EGF-containing fibulin-like extracellular matrix protein 1, Myoglobin, POTE ankyrin domain family member I, Actin, cytoplasmic 2, Lysozyme, ComplementC1qC&B, Ig κ variable 3D-15&3-20, IgGFcbinding protein, Vitronectin, α-2- macroglobulin.
Asano 2011	Japan	Paediatric acute encephalopathy vs. febrile seizures	13 vs. 42	Biomarker Discovery: 1) CSF from 8 cases vs. 28 controls was applied to a 96-well Protein Chip and analysed by SELDI-ToF MS to identify differences in peaks; 2) This was repeated in a different group of samples (referred to as validation) with 5 cases vs. 14 controls; 3) The peak with the best separation between the groups was analysed by tandem MS (MALDI-QSTAR) to enable protein identification.	CSF	SELDI-TOF and MALDI-QSTAR	Human	Identification with Mascot, quantification and statistical analysis with Protein Chip Data Manager software	Kruskall-Wallis H test, Mann-Whitney test with Bonferroni-Dunn correction (P value <0.05).	1 host protein differentially expressed in between cases vs. controls: Peptide fragment from the neurosecretory protein VGF precursor. Note – 15 peaks were different in the first experiment, and of these 4 were confirmed as being different in the second, but only one of these was subject to tandem MS to enable protein identification.
Bonnet 2018	France	*T.b. gambiense* early stage vs. late stage vs. controls (CSF <5 WCCs/µl and no trypanosomes)	3 vs.4 vs.3	Biomarker Discovery:1) CSF from 2 case groups vs. controls (3, 4, 4) compared by LC-MS, involving abundant protein depletion and concentration by filtration, and then analysed by label-free LC-MS. Biomarker Verification: A larger group (23, 43 vs. 14) used to verify potential biomarkers using label-free LC-MS and then ELISA to detect neuroserpin, neogenin and secretogranin 2.	CSF, urine and saliva.	Q Exactive Orbitrap	Humans and Pathogen	Identification with Proteome Discoverer, Mascot, quantification and statistical analysis with Progenesis QI.	ANOVA (P value <0.05)	37 host proteins differentially expressed between groups and with a biological role^e^. 3 host proteins were selected for verification: Neuroserpin, neogenin and secretogranin 2;only 1 host protein, neuroserpin, was detected by ELISA, and this differentiated between early and late stage disease, with AUROC 0.72, sensitivity 94% and specificity 58%.
Cordeiro 2015	Brazil	Pneumococcal vs. Meningococcal vs. Enterovirus meningitis	3 vs.3 vs.3	Biomarker Discovery: 1) CSF from 6 of each of the 3 types of meningitis and 6 controls (patients without infection, neurodegenerative or psychiatric disease) tested involving abundant protein depletion and then separation with 2D gels; 2) 3 patients from each type of meningitis selected for running as pooled samples on gel, gel-extraction and MS.	CSF	MALDI-ToF	Human	Identification with Mascot	Qualitative analysis - proteins present in 11/12 gels per group, and not present in another group	4 host proteins differentiated between groups and were used to develop a predictive model of meningitis: Apolipoprotein A-I (present in all causes of meningitis and not controls), C-reactive protein & Complement C3 (present in bacterial meningitis and not viral), Kininogen-1 (present in Meningococcal meningitis).
Fraisier 2014	France	West Nile virus vs Non-WNV infection, headache, idiopathic intracranial hypertension and healthy controls.	8 vs.11	Biomarker Discovery: 1) CSF from 8 pooled cases vs. 11 pooled controls (6 with acute headache and 5 with idiopathic intracranial hypertension) analysed using iTRAQ labelling, isoelectric point based separation into 12 fractions and then LC-MS analysis. Biomarker Verification: Secondly, CSF and/or serum tested for the identified protein Defensin Alpha-1 by an ELISA in 16 cases vs. 13 controls.	CSF and Serum	LTQ	Human	Identification, quantification and statistical analysis with Mascot and SEQUEST through Proteome Discoverer.	Kruskal-Wallis and Mann-Whitney U tests, P value <0.05	47 host proteins differentially expressed in cases vs controls during discovery. 1 host protein, Defensin α-1, was selected based on its high fold-change, possible functional association with WNV pathobiology, potential use as severe infection biomarker, and availability of commercial ELISA tests.
Gomez-Baena 2017	UK	Pneumococcal meningitis vs controls (normal CSF)	16 vs. 12	Biomarker Discovery: 1) CSF from 16 cases vs. 12 controls analysed label-free using LC-MS; 2) CSF from 20 cases and 15 controls used to confirm a subset of proteins by western blotting.	CSF	LTQ-Orbitrap Velos	Human and Pathogen	Identification with Proteome Discoverer and Mascot, quantification and statistical analysis with Progenesis QI.	ANOVA, P value <0.05	134 host proteins and 6 Streptococcus proteins differentially expressed in cases vs controls during discovery. 5 host proteins were selected and confirmed by western blotting based on confidence of protein identification, magnitude of change, extent of protein coverage and putative role in the pathology: Myeloperoxidase, S100 calcium binding protein A9, Cathelicidin antimicrobial peptide, Ceruloplasmin and Cystatin C.
Mu 2015^c^	China	Tuberculous meningitis vs healthy controls	12 vs. 12	Biomarker Discovery: 1) CSF from 12 cases vs. 12 healthy controls analysed, including high abundant protein depletion, protein digestion, iTRAQ labelling, SCX fractionation, and LC-MS. Biomarker Verification^d^: CSF from 25 cases and 28 controls tested for Apolipoprotein B (ApoB) and Apolipoprotein E using ELISA.	CSF	Triple-ToF	Human	Identification, quantification and statistical analysis with ProteinPilot.	Not reported, P value <0.05.	81 host proteins differentially expressed in cases vs controls. 2 host proteins were selected for their role in lipid metabolism and confirmed by western blotting and ELISA: Apolipoprotein B & E. Apo B was confirmed, with AUROC 0.91, sensitivity 89% and specificity 92%.
Njunge 2017	Kenya	Acute Bacterial Meningitis vs. Cerebral Malaria	37 vs. 22	Biomarker Discovery: CSF from 37 ABM cases vs. 22 CM cases was analysed label-free using LC-MS.	CSF	Q Exactive Orbitrap	Human	Identification, quantification and statistical analysis with MaxQuant and R.	Mann Whitney test, P value <0.05, AUROC >0.9	52 host proteins differentially expressed in the two groups. 2 host proteins were identified with sensitivity >98% and specificity = 1: Myeloperoxidase and Lactotransferrin
Ou 2013	China	Tuberculous meningitis, Cryptococcal meningitis vs. Healthy controls	20 vs. 20 vs. 20	Biomarker Discovery: CSF from 20 pooled TBM vs. 20 pooled Crypto cases vs. 20 pooled controls, fractionated using SCX chromatography and analysed by LC-MS. Biomarker Verification^d^: CSF from 25 cases vs. TBM, 25 Crypto vs. 25 controls tested for S100A8 and Apo Busing ELISA.	CSF	QSTAR	Human	Identification, quantification and statistical analysis with ProteinPilot.	Not reported, P value <0.05.	9 host proteins differentially expressed in cases vs controls. 2 host proteins were selected for and confirmed by western blotting and ELISA: Apolopoprotein B and S100 calcium binding protein A8
Sengupta 2015	India	Japanese encephalitis virus vs. Non-JEV Acute Encephalitis Syndrome	10 vs. 10	Biomarker Discovery: CSF from 10 pooled cases and 10 pooled controls separated by SDS-PAGE, JE-specific visualised spots excised and analysed by MS.	CSF	MALDI-ToF	Human	Identification, quantification and statistical analysis with ProteinPilot using MASCOT.	Qualitative analysis of proteins visualised only in JEV cases	7 host proteins identified only in cases: Serum albumin, Vitamin D-binding protein, Fibrinogen gamma chain, Fibrinogen beta chain, Complement C3 & C4b, Actin cytoplasmic-1.
Tiberti 2015	Switz-erland	*T. bruceii gambiense, T. rhodesiense* vs. controls (CSF <5 WCCs/μl and no trypanosomes)	3 vs. 3	Biomarker Discovery: 1) CSF from 3 cases of T. rhodesiense vs. 3 cases of T gambiense labelled with TMT, isoelectric point based separation into 12 fractions and analysed by MS. Biomarker Verification: CSF of 39 and 126 cases, and from 20 non-infected control subjects. In addition to CSF, n = 29 plasma samples obtained from 15 and 14 cases.	CSF and Plasma	LTQ Orbitrap Velos	Human and Pathogen	Identification, quantification and statistical analysis with EasyProt.	Kruskall-Wallis H test, Mann-Whitney test with Bonferroni-Dunn correction (P value <0.05).	11 host proteins differentially expressed between groups. 3 proteins were selected for further verification, based on their TMT ratios as well as on their appearance in the pathways of interest: C-reactive protein, Orosomucoid-1 and Complement component 9.

a Site refers to the location of the mass spectrometry work.

b Sample sizes presented include the number of patient CSF samples tested by mass-spectrometry methods, cases vs. controls.

c Yang *et al* 2015 is not presented as it duplicates the work reported by Mu *et al* 2015.

d Reported as validation, however this is verification.

e Only CSF biomarkers are reported.

There were a range of infections studied, including bacterial, viral and parasitic infections (Table 2). Additional corresponding samples to CSF were analysed in three studies, two articles also described analysis of serum and another included plasma. Importantly, the most recent study was the only one to analyse additional non-invasive samples, saliva and urine.^53^ Non-invasive samples for biomarker detection are likely to be of great importance for improving diagnostic capacity in clinical practice.

The method of collecting CSF was not described in any article. Eight (73%) articles reported the method of storage, of which four (36%) involved centrifugation, and two (18%) involved snap freezing with liquid nitrogen. Three (27%) studies used immunodepletion to remove highly abundant proteins such as albumin and transferrin. Ten (91%) studies fractionated the samples, using various combinations of offline and MS coupled systems. One (9%) involved gel-extraction methods, while the others were gel-free. Four (36%) used labelling, using isobaric tags for relative and absolute quantitation (iTRAQ) or tandem mass tags (TMT). One study reported the use of an internal control, bovine beta-lactoglobulin.^50^ All studies involved bottom-up MS approaches. Three (27%) studies used time-of-flight mass-spectrometers, and the other eight (73%) used quadrupole mass-spectrometers. Extracted data was searched using human proteome databases for all, and four also searched using pathogen databases.

At the discovery stage, a median (range) of 13 (1–140) potential biomarkers were identified per study. A sub-group of six studies performed further evaluation, using either targeted MS or antibody-based confirmation, confirming findings in a median (range) of 2 (1–5) proteins. Three studies referred to an additional study group as verification, although two of these studies interchangeably used the term validation for the same analysis.^43^, ^53^ The aim of the verification stage is to confirm numerous potential biomarker(s), and reduce the numbers down to a single marker or combination (<10) markers that may be feasible to subsequently test in an antibody-based platform. The sample size for verification is 50–200, and the method of detection is usually targeted MS. One study incorporating verification used targeted MS, while the other two studies reporting verification used ELISA assays to confirm 1–3 biomarkers. Similarly, three studies referred to validation involving ELISA assays to confirm biomarkers in samples of 25–66 cases. There was no sample size calculation reported to corroborate the results.

Eight (73%) studies reported investigation of pathway analysis, functional clustering and/or subcellular localisation, with programs such as the Gene Ontology (GO) tool,^54^ and STRING.^55^ Four (36%) uploaded the data to an open-access database, such as PRIDE (http://www.ebi.ac.uk/pride).^56^ All the biomarkers identified await further evaluation, and implementation as diagnostic tests. No follow-up publications were found for any of the included studies, or from personal correspondence with authors.

It is noteworthy that only one (25%) study investigating pathogen-derived biomarkers identified any,^44^ suggesting low sensitivity of these techniques for pathogen-derived proteins.^40^ The biomarkers identified included plasma proteins associated with damage to the blood-brain barrier, immune activation, inflammation, and proteins from brain tissue associated with parenchymal damage (summarised in Table 2). Notably, both studies on *Mycobacterium tuberculosis* infection identified apolipoprotein B, although one also identified apolipoprotein E and the S100 calcium binding protein A8.^45^, ^47^ Similarly, both studies involving *Streptococcus pneumoniae* infection identified myeloperoxidase, although one also identified lactotransferrin, and the other identified S100 calcium binding protein A9, cathelicidin antimicrobial peptide, ceruloplasmin, and cystatin C.^42^, ^44^ The two studies investigating *Trypanosoma* sp. identified different biomarkers, C-reactive protein and ORM1, and neuroserpin.^50^, ^53^

## Discussion

To the best of our knowledge, this is the first systematic review of the application of proteomics-based MS for identification of clinical biomarkers for diagnosing CNS infections. We highlight an emerging field, currently largely limited to pre-clinical discovery studies. This is not surprising when you consider that the human proteomics project was begun over a decade after the human genomics project, and proteomics investigation has lagged behind that of genomics.^57^

Eleven studies were identified that met the eligibility criteria, included in the final quality assessment and data extraction, of which eight (73%) were published within the last five years. Eleven pathogens were studied, and a median (range) of 13 (1–140) potential protein biomarkers identified per study. There was some consistency in biomarkers identified in different studies of the same pathogen. However, none of the biomarkers have been further investigated in subsequent publications or commercialised. It is unclear if this work is currently ongoing, not planned due to a lack of infrastructure and investment to carry the biomarkers through to validation, or it has happened but has not been published due to poor performance in further testing.

Successful application of MS depends on appropriate samples and well-designed experiments.^58^ For this, interdisciplinary collaboration is key, and the process is a significant undertaking. It is not sufficient for samples to be deposited with a MS laboratory, awaiting output of candidate biomarkers. To fully harness the technology, there need to be clear research questions and criteria for a potential biomarker. In line with this, and providing source for improvement in future studies, quality assessment revealed sources of bias in study design, such as a lack of clear case definitions for the target diseases and controls, explicit sampling methodology, sample size and statistical methods. In particular, there were inconsistencies in matching between cases and controls.^59^ It is possible that this may partially be explained by standards of reporting, as even though further details were requested, there was a low response rate (2/11, 18%) to these requests. However, it is also clear that the experiments were frequently conceived retrospectively, using samples remaining from various other studies. The findings also emphasise the challenges of conducting these studies, due to limitations in obtaining sufficient CSF samples, or reaching a consensus on case definitions. Although three of the studies reported validation this was not considered validation due to the small sample size and lack of a sample size calculation. None of the studies took further steps to investigate impact in clinically relevant groups, such as consecutive patients with suspected CNS infections.

While all studies reported that ethical approval was obtained, it is important to recognise the subtle differences in permission that should be sought for a study on biomarker discovery. For example, an unbiased biomarker discovery study may identify a disease in the participant that is unrelated to the project aim. Another ethical issue is the use of CSF from healthy people, as CSF must be obtained by a significantly invasive procedure that does not have zero risk. Three studies involved obtaining CSF samples from healthy participants with informed consent, that may not be deemed ethical in all countries. Healthy CSF is not necessary, as an appropriate control group would be other CNS infections.^34^ Additionally, commercial samples marketed as healthy CSF need to be utilised cautiously as clinical characterisation and diagnostic testing may not be comprehensive, or fully disclosed or ethical.

Sample preparation and mass spectrometry analysis are dependent on the volumes of CSF and equipment available. The search for biomarkers has been equated to the search for a needle in a haystack.^60^ The optimal strategy for probing CSF proteins remains elusive due to the high variability in methods used, i.e. there are few studies that systematically investigate the effect of changing one factor in the long series of experiments required to perform LC-MS protein analysis.^61^ It is clear that a small number of proteins such as albumin and transferrin are highly abundant. Nonetheless, strategies of immunodepletion require large volumes of CSF, and may also remove proteins of interest bound to abundant ones. To this end, only three (27%) studies performed highly abundant protein depletion. It is unclear if in-gel digestion is superior to in-solution digestion. In-gel digestion would lose smaller <10 kDa proteins. However, there are few relevant proteins of this size, and the impact is estimated to be minimal. Similarly, filtration devices used to concentrate CSF are also implemented, however these may lead to protein losses, and not only proteins below the molecular weight cut-off of the device. Although, the standard enzyme used for digestion is trypsin, neuropeptides do not terminate in predictable in amino-acids, and have more post-translational modifications such as glycosylation and phosphorylation. For this reason, the use of Lys-C has been used to improve protein fragmentation.

In the review, four (36%) studies used isobaric mass-tagging to allow simultaneous analysis of more than one sample in the same mass-spectrometry experiment. This is beneficial as it reduces bias due to the inherent variability between runs, improves relative quantification between samples, speed and equipment costs. Nonetheless, the larger number of steps required during sample preparation may lead to protein losses, and tagging methods are more expensive. Label-free quantification is steadily improving. The strategy for separation is very important, and a wide variety of methods are used, both off-line and online (coupled to the MS machine). It is clear that multidimensional orthogonal strategies are necessary, i.e. sequential methods of separation involving different mechanisms such as separation by mass and charge. For instance, a useful strategy has been strong cation exchange coupled with online reverse-phase LC-MS. Different strategies, including diverse series of sample preparation methods, and protein identification software may reveal different biomarkers.^61^ For this reason, if there is sufficient CSF it may be useful to try alternative methods.

Notably, while all studies searched human proteome databases, only four studies also searched pathogen-derived databases. It is possible that researchers were convinced that pathogen searches would be futile. However, for the purposes of diagnostics, pathogen-derived biomarkers are likely to be more specific than host biomarkers, and it would seem sensible to attempt to look for them during the analysis.

Limitations of this review include the restriction to mass-spectrometry proteomics analysis, English language, non-HIV infection and human CSF. HIV infection provides an additional level of complexity due to the high incidence of opportunistic infections in this disease. Studies of animal model CSF may also provide informative data. Publications involving mass-spectrometry that did not involve unbiased discovery work, ‘peptide mass fingerprinting’ or ‘peptide sequencing’, for instance MALDI-TOF without protein sequencing, were not included. Additionally, the review did not extend to transcriptomics, post-translational-omics or metabolomics research.^62^

The discussion has focussed on pre-clinical biomarker discovery, as this has been the focus of studies to date, but clinical validation is an essential part of the process. Immunoassays are more sensitive and easier to use, but have their own problems related to non-specific reactivity.^63^, ^64^ A validation study needs a sample size calculation, requiring approximately 100–1000 patient samples, tested in hospital patients, and needs comparison with the gold-standard diagnostic test. The results would need to be presented as sensitivity, specificity, positive, and negative predictive value, with ROC curve analysis and area under the ROC (AUROC) values. These studies require considerable investment. Until these aspects are acknowledged, and studies designed accordingly, it is unlikely that biomarker discovery will be successfully translated to the bedside.

The systematic application of MS methods to identify protein biomarkers for specific infections, or groups of infections, holds promise to improve diagnostic yield in the challenging group of CNS infections that remain undiagnosed. Furthermore, strengthening knowledge of these proteins will also inform understanding of pathophysiology of infection, and identify targets for developing therapeutics.

## Contributors

TB conceived the study. PN, NZ, BG, AK, ADP, and MW helped to refine the study design and develop the protocol. TB performed the initial literature search, and together with BG assessed the studies according to the inclusion criteria, performed the quality assessment and data extraction. AK acted as a third assessor to resolve disagreements. TB wrote the initial draft of the manuscript, and all the authors contributed to editing and developing the article for submission.

## Declaration of Competing Interest

None of the authors have any conflict of interests to report.

## Appendix Supplementary materials

Image, application 1

Image, application 2
