# Supplementary material for: Mass spectrometry-based proteomic techniques to identify cerebrospinal fluid biomarkers for diagnosing suspected central nervous system infections. A systematic review
Source: J Infect. 2019 Nov;79(5):407–18. doi: 10.1016/j.jinf.2019.08.005 (PMC6838782; doi:10.1016/j.jinf.2019.08.005)
Supplement: Supplementary file 1 [file mmc1.docx]

**Supplementary Data A: Quality Assessment of Included Studies across Eight Criteria as per Mischak *et al***

| **Study** | **1. Describe and justify the clinical question, outcomes, and selection of subjects.** *Describe the clinical question and justify why it is of interest; describe what outcomes are assessed and comment on their clinical validity, potential for misclassiﬁcation, and veriﬁcation bias, if pertinent; clarify what are the eligibility criteria for the selected study populations and justify speciﬁc choices.* | | **2. Describe the assessed subjects.** *Provide demographic information with gender, age, ethnic origin, and concomitant medications at a minimum, and all relevant disease-related and clinical parameters.* | | **3. Describe sampling.** *Accurate description of the sampling conditions and procedures (including the collection process and any manipulation of the sample before storage, the time between sampling and storage, storage conditions, and the addition of any protease inhibitors +/- preservatives). Justify sampling according to the literature or supporting experimental data.* | | **4. Describe experimental methodology.** *Procedure, as well as observed SD of related technical speciﬁcations. To attribute the same identity to a certain feature in several independent analyses, report accepted deviations of mass and other parameters (retention time, migration, position on gel, etc.). Also, report the observed deviation in identifying parameters and (relative) abundance, when re-analysing the same sample.* | |
| --- | --- | --- | --- | --- | --- | --- | --- | --- |
|  | *Y/P/N* | *Notes* | *Y/P/N* | *Notes* | *Y/P/N* | *Notes* | *Y/P/N* | *Notes* |
| Angel (2012) | Partially | Reported aim is to study 'the broad spectrum of changes in proteins bathing the CNS following early-disseminated Lyme disease'; however, the background and potential clinical question is not explained. Discussion of positive and negative controls. No explanation as to why a patient without CSF pleocytosis is included as a case. Equally, for the controls, No report of whether they had Lyme disease. | Partially | Reporting of the age, sex and some clinical presentations, but not the ethnicity, comorbidities, medications, BMI, or physiological parameters. | Partially | Reporting of the sample preparation, storage and transport, but not the time of storage. | Yes | Reporting of the sample preparation and mass-spectrometry experiments |
| Asano (2011) | Partially | Clear question, and potential clinical role of a diagnostic biomarker (although No discussion of what the potential intervention might be if the biomarker did manage to discriminate between acute encephalopathy and febrile seizures). Limited reporting of inclusion criteria, such as the dates and method of sampling. | Partially | Limited reporting of details of the cases or controls. | Partially | Reporting of the sample preparation and storage, but not the time of storage. | Yes | Reporting of the sample preparation and mass-spectrometry experiments |
| Bonnet (2018) | Yes | Clear question, with reporting of potential clinical impact, case definitions and study inclusion criteria. | Partially | Reporting of age and sex, but not ethnicity, clinical presentations, comorbidities, medications, BMI, or physiological parameters. | Partially | Reporting of the storage, but not full details of sample collection and the time of storage. | Yes | Reporting of the sample preparation and mass-spectrometry experiments |
| Cordeiro (2015) | Partially | Clear question, and potential clinical role of a diagnostic biomarker. Limited reporting of details of the inclusion criteria, such as the dates and method of sampling. | Partially | Reporting of age, sex and ethnicity, not clinical presentations, comorbidities, medications, BMI, or physiological parameters. | Partially | Reporting of the sample preparation and storage, but not the time of storage. | Yes | Reporting of the sample preparation and mass-spectrometry experiments |
| Fraisier (2014) | Partially | Clear question, and potential clinical role of a diagnostic biomarker. No reporting of details of the inclusion criteria, such as the dates and method of sampling. | Partially | Reporting of age, sex and ethnicity, but not clinical presentations, comorbidities, medications, BMI, or physiological parameters. | No | Unclear where the samples were collected, or how they were stored and transported. | Yes | Reporting of the sample preparation and mass-spectrometry experiments |
| Gomez-Baena (2017) | Yes | Clear question and reporting of potential clinical impact. | Partially | For cases, reporting of age, sex and ethnicity, but not clinical presentations, comorbidities, medications, BMI, or physiological parameters. Aside from ethnicity, none reported for the controls. | Partially | Reporting of the sample preparation and storage, but not the time of storage or how they were transported. | Yes | Reporting of the sample preparation and mass-spectrometry experiments |
| Mu (2015) | Partially | Clear question and reporting of potential clinical impact. Limited reporting of the study inclusion criteria, and risk of verification bias. | No | Not reported (Reported patients samples that underwent ELISA, and it is not clear if some of these were also used in MS experiments, nonetheless the data cannot be extracted). | No | Not reported | Yes | Reporting of the sample preparation and mass-spectrometry experiments |
| Njunge (2017) | Partially | Clear question, and potential clinical role of a diagnostic biomarker (although No discussion of what the potential intervention might be if the biomarker did manage to discriminate between acute encephalopathy and febrile seizures). Limited reporting of details of the inclusion criteria, such as the dates and method of sampling. | Partially | Reporting of age, sex, but not ethnicity, clinical presentations, comorbidities, medications, BMI, or physiological parameters. | No | Not reported | Yes | Reporting of the sample preparation and mass-spectrometry experiments |
| Ou (2013) | Partially | Clear question, with reporting of potential clinical impact and case definitions. Limited reporting of the study inclusion criteria. | Partially | Reporting of age, sex, but not ethnicity, clinical presentations, comorbidities, medications, BMI, or physiological parameters. | No | Not reported | Yes | Reporting of the sample preparation and mass-spectrometry experiments |
| Sengupta (2015) | Partially | Clear question, with reporting of potential clinical impact and case definitions. Limited reporting of the study inclusion criteria. | Partially | Reporting of age, sex, but not ethnicity, clinical presentations, comorbidities, medications, BMI, or physiological parameters. | Partially | Reporting of sample collection, but not preparation, storage, time of storage or how they were transported. | Partially | Reporting of the sample preparation and mass-spectrometry experiments, but this would not be reproducible |
| Tiberti (2015) | Yes | Clear question, with reporting of potential clinical impact, case definitions and study inclusion criteria. | Partially | Reporting of age, sex, but not ethnicity, clinical presentations, comorbidities, medications, BMI, or physiological parameters. | Partially | Reporting of the sample preparation and storage, but not the transport. | Yes | Reporting of the sample preparation and mass-spectrometry experiments |

| **Study** | **5. Describe the statistical evaluation.** *Provide details on sample size, statistical analysis plan (for appraising calibration, discrimination, and/or reclassiﬁcation), any consideration or adjustment for covariates (including treatment, whenever pertinent), methods for adjustment for multiplicity, and parameters used in complex machine-learning approaches, whenever pertinent. Clarify which analyses are predeﬁned and which are post hoc.* | | **6. Validate results.** *The results must be conﬁrmed in at least one independent sample set. The sampling and characteristics of the validation population should be reported, and the analysis should be symmetrical in the test and validation data sets; any deviations should be reported.* | | **7. Acknowledge limitations.** *No study is perfect; limitations should be clearly acknowledged and their potential impact on the results discussed. Including sources of potential bias, statistical uncertainty, and generalisability.* | | **8. Take responsibility.** *The contributions of each author should be clearly stated.* | |
| --- | --- | --- | --- | --- | --- | --- | --- | --- |
|  | *Y/P/N* | *Notes* | *Y/P/N* | *Notes* | *Y/P/N* | *Notes* | *Y/P/N* | *Notes* |
| Angel (2012) | Partially | No mention of the rational in samples size. The statistical calculations were reported in the results section, but not in the methods. | No | No validation performed. | Partially | Discuss limitation that the controls had not all been tested for Lymes disease, but no comment on the fact that all the cases did not fulfil their case definition, or that the study was performed in a small sample. No mention of ethnicity, or the difference in age of cases and controls. | No | Not stated |
| Asano (2011) | Partially | No mention of the rational in samples size. The statistical calculations were reported in the results section, but not in the methods. | No | No validation performed. | No | No discussion of the limitations. One of the most important is the small sample size, with only 8 cases in group 1, and 5 in group 2. | No | Not stated |
| Bonnet (2018) | Partially | No mention of the rational in samples size. The statistical calculations were reported. | Partially | Verification using ELISA performed in a small sample, however no validation performed. | Yes | Report potential limitations in the discussion | No | Not stated |
| Cordeiro (2015) | No | No mention of the rational in samples size, or details of statistical calculations. | No | No validation performed. | No | No discussion of the limitations. One of the most important is the small sample size, with only 6 cases in each group. | Yes | Clearly stated. |
| Fraisier (2014) | Partially | No mention of the rational in samples size. The statistical calculations were reported. | Partially | Verification using ELISA performed in a small sample, however no validation performed. | No | No discussion of the limitations. | Yes | Clearly stated. |
| Gomez-Baena (2017) | Partially | No mention of the rational in samples size. The statistical calculations were reported. | No | No validation performed. | No | No discussion of the limitations. | Yes | Clearly stated. |
| Mu (2015) | Partially | No mention of the rational in samples size. The statistical calculations were reported. | No | No validation performed. Confirmation using ELISA, however unclear if this includes the same patients as the MS experiments, and involves only 40 cases. | No | No discussion of the limitations. | No | Not stated |
| Njunge (2017) | Partially | No mention of the rational in samples size. The statistical calculations were reported. | No | No validation performed. | Yes | Report potential limitations in the discussion | No | Not stated |
| Ou (2013) | Partially | No mention of the rational in samples size. The statistical calculations were reported. | No | No validation performed. Confirmation using ELISA, however unclear if these are the same patients as used in MS experiments, and involves only 25 and 25 cases. | No | No discussion of the limitations. | No | Not stated |
| Sengupta (2015) | Partially | No mention of the rational in samples size. The statistical calculations were reported. | No | No validation performed. | No | No discussion of the limitations. | Yes | Clearly stated. |
| Tiberti (2015) | Partially | No mention of the rational in samples size. The statistical calculations were reported. | No | Verification but not validation performed. | Yes | Report potential limitations in the discussion | Yes | Clearly stated. |
