# Supplementary material for: Mass spectrometry-based proteomic techniques to identify cerebrospinal fluid biomarkers for diagnosing suspected central nervous system infections. A systematic review
Source: J Infect. 2019 Nov;79(5):407–18. doi: 10.1016/j.jinf.2019.08.005 (PMC6838782; doi:10.1016/j.jinf.2019.08.005)
Supplement: Supplementary file 2 [file mmc2.docx]

**Supplementary Table B: Data Extraction of Included Studies**

| **Study** | **Site of Mass Spectrometry** | **Type of Study^a^**  *Discovery/ verification/ validation* | **Participants^b^**  *Details of recruitment (setting, location and dates; whether sampling was consecutive, random or convenience series)* | **Target Condition or Syndrome** |
| --- | --- | --- | --- | --- |
| Angel (2012) | USA | i. Discovery | Patients at either Massachusetts General Hospital or Maine Medical Centre (U.S.A.) attending 2006-09 for investigation of neurological symptoms associated with Lyme disease for whom an LP was performed. Controls involved a different population; patients who had an LP performed for a variety of clinical indications. Details of sampling are not reported. | Early-disseminated Lyme disease (*Borrelia burgdorferi sensu stricto* infection) and CSF inflammation. |
| Asano (2011) | Japan | i. Discovery  ii. Further work reported as validation | Patients at the Department of Paediatrics, Nippon Medical School Chiba Hokusoh Hospital (Japan) with encephalopathy or febrile seizures. At least two paediatric neurologists confirmed the diagnosis based on clinical findings, routine examinations, and LP performed. It is reported that some of the patients with encephalopathy had infectious aetiologies confirmed, but it is not clear if this was the case for all patients. Details of dates and sampling are not reported. | Paediatric acute encephalopathy |
| Bonnet (2018) | France | i. Discovery  ii. Further work reported both as verification and validation | The samples used in this study were collected during a prospective cohort study carried out 2009-2011 in three provinces (Angola). The study enrolled 247 Angolan subjects testing positive for human African trypanosomiasis (HAT) by the card agglutination test (CATT). Every subject was examined clinically and submitted to a questionnaire to document clinical and neurological characteristics. Neurological data included an index of depression, measured with the Hamilton rating scale, and the sleep and psychiatric disturbance using the Mini-International neuropsychiatric interview. Main coinfections were excluded in the field by microscopy for common parasitic coinfections (blood smear for malaria, CTC for detection of filariasis, examination of a urine sediment for schistosomiasis when urine was positive for blood). Retrospectively, samples were tested for HIV and syphilis, and all positive samples were excluded from the study. | *Trypanosoma bruceii gambiense (T.b.gambiense)* - Early vs. Late Stage |
| Cordeiro (2015) | Brazil | i. Discovery | Patients admitted at Hospital Giselda Trigueiro or Children’s Hospital João Paulo II FHEMIG (Brazil). Details of dates and sampling are not reported. | Pneumococcal, Meningococcal vs Enterovirus meningitis. |
| Fraisier (2014) | France | i. Discovery  ii. Further work reported both as verification and validation | Patients with stored CSF available (Greece, the Netherlands, France). Details of dates and sampling are not reported. | West Nile virus neuroinvasive disease (WNND) |
| Gomez-Baena (2017) | UK | i. Discovery | Patients at Queen Elizabeth Central Hospital in Blantyre (Malawi) April 2004 to October 2006. Further details in citation; Children aged 2 months to 16 years identified as a possible case of pneumonia (respiratory rate ≥50 min−1 for children <12 months and ≥40 min−1 for children ≥12 months) or meningitis (stiff neck, bulging fontanelle, fever and convulsions) were recruited into the study. | Paediatric pneumococcal meningitis |
| Mu (2015) | China | i. Discovery  ii. Further work reported as validation | Patients at enrolled in the Department of Neurology, First Affiliated Hospital of Chongqing Medical University (China), were enrolled. Details of dates and sampling are not reported. | Tuberculous meningitis (TMB) |
| Njunge (2017) | Kenya | i. Discovery | Paediatric admissions (2002–11) at Kilifi County Hospital (Formerly Kilifi District Hospital), Kilifi (Kenya). Details of sampling are not reported. | Acute bacterial meningitis (ABM) and Cerebral Malaria |
| Ou (2013) | China | i. Discovery  ii. Further work reported as validation | Patients seen by the Department of Infectious Diseases of Shanghai Public Health Clinical Centre (China) January 2009 to December 2010 and healthy volunteers. Details of sampling are not reported. | Tuberculous meningitis (TBM) and Cryptococcal meningitis |
| Sengupta (2015) | India | i. Discovery | Patients admitted to King George’s Medical University in Lucknow, Uttar Pradesh (India) with acute encephalitis syndrome (AES) or Japanese encephalitis virus infection (JE). Inclusion criteria for LP: i) age >3 years but excluding pregnant women, ii) presence of fever with altered sensorium of 7 days or less. Exclusion criteria were: i) a firm alternative etiological diagnosis, ii) some contraindication to drug administration. Details of dates and sampling are not reported. | Japanese encephalitis virus infection (JE) |
| Tiberti (2015) | Switzerland | i. Discovery  ii. Further work reported as verification | Refers to a previous publication; Patients were identified through active and passive case finding between 2005 and 2009 (Angola, Chad and the Democratic Republic of the Congo). Sample collection was done in the context of several prospective diagnostic studies, or was directed by the WHO for a HAT specimen bank. | *T.b.gambiense* vs T.b.*rhodesiense* |

^a^Several studies reported further work as verification or validation, however none of these involved a priori sample size calculations. ^b^All studies were retrospective.

| **Study** | | **Case Definition**  *Inclusion criteria for cases; discussion of whether this is a reference standard or combination of factors, details of the reference standard and interpretation of results, and the rational for these)* | | | | | | | | | | | **Number of Cases** | | | **Cases - % Females** | | | **Cases - Median Age** | | | **Cases - Ethnicity** | | | **Cases – Comorbidities** *Pregnancy, epilepsy* | | | | | **Cases - Medications** | | | |
| --- | --- | --- | --- | --- | --- | --- | --- | --- | --- | --- | --- | --- | --- | --- | --- | --- | --- | --- | --- | --- | --- | --- | --- | --- | --- | --- | --- | --- | --- | --- | --- | --- | --- |
| Angel (2012) | | CDC guidelines 2011. CSF pleocytosis (>5 WBCs/mm3). However out of 26 patients, 22 met the criteria and 4 were included based on clinical and laboratory data. | | | | | | | | | | | 26 | | | 35% | | | Mean age 27 (4-67) | | | Not explicit | | | Not reported | | | | | Not reported | | | |
| Asano (2011) | | 1) impaired consciousness, 2) signs of increased intracranial pressure due to brain oedema, 3) convulsions and seizures, 4) slow activity on electroencephalography lasting more than 24 h after acute onset, and 5) no bacteria or fungi on CSF culture. Reference cited: Johnston MV: Acute encephalopathies. In Nelson Textbook of Paediatrics. | | | | | | | | | | | 13 (8 discovery, 5 ‘validation’) | | | 46% | | | Mean age months 46.4 ± 33.4; and 48.4 ± 38.5. | | | Not explicit | | | Not reported | | | | | Not reported | | | |
| Bonnet (2018) | | CATT test positive and Trypanosomes detected in a body fluid; Late = CSF WCC <5 and Trypanosomes not detected. | | | | | | | | | | | 3, 4. Subsequently 66 ELISA | | | 0% | | | Mean age 43.3 (SD 23.1), 31.3 (18.1) | | | Not explicit | | | Not reported | | | | | Not reported | | | |
| Cordeiro (2015) | | Patients with confirmed meningitis. Pneumococci, or meningococci in CSF, or blood specimens detected by culture, Gram staining, and/or latex agglutination test. CSF white blood cell counts greater than 100 cells/mm3 being more than 50% neutrophils. Patients with enteroviral meningitis had pleocytosis characterized mostly by mononuclear cells in the absence of detectable bacterial pathogen and enterovirus RNA detected. One patient with viral meningitis had mild pleocytosis, no meningeal signs at LP, but tested positive for Enterovirus RNA. | | | | | | | | | | | 6, 6, 6 | | | 17%, 67%, 50% | | | 17.5 (2 - 36), 9.5 (<1 - 13), 6.0 (<1 - 8) | | | Yes. N-E vs S-E. 67%, 17%, 0% | | | Not reported | | | | | Not reported | | | |
| Fraisier (2014) | | Not reported. In the discussion it is mentioned that detection of IgM is the standard for diagnosis of WNV, however it is not clear if this was the definition. A1) CSF and serum, A2) only CSF, A3) only serum, and patients A2 with only CSF were used in the MS experiments. Note after the MS experiments, the other groups A1 and A3, and fourth group of cases B1 of 'WNV fever' patients serum samples were evaluated using an ELISA. | | | | | | | | | | | 8. Subsequently 33 (16,27) ELISA | | | 49% (56.3, 62.5, 40.7). 62.5. | | | 64.8 (19–80) 71.2 (42–87) 68.7 (44–92). 71.2 (42-87) | | | Yes. Caucasian. Greek. | | | Not reported | | | | | Not reported | | | |
| Gomez-Baena (2017) | | Confirmation of acute bacterial meningitis (ABM) was defined by a child presenting symptoms of ABM, a CSF cell count > 10/mm3 and one of the following tests for *Streptococcus pneumoniae* positive: CSF culture, Gram stain, polysaccharide antigen or PCR. All samples were cultured on sheep blood and chocolate agar for 48 h under aerobic and microaerophilic conditions. Bacteria were identified using standard methods53. Pneumococcal bacterial DNA was amplified and quantified using a real-time PCR assay. | | | | | | | | | | | 8,8. Subsequently 20 confirmed by gel-based methods. | | | 63% | | | 1.0 (0.5- 4.9) | | | Yes. Malawian | | | Not reported | | | | | Not reported | | | |
| Mu (2015) | | Cited reference (Ahuja *et al*, 1994) for the 'standard definition' of TBM, but there is not report about whether the cases had definite, highly probable, probable or all three. | | | | | | | | | | | 12. Subsequently 25 ELISA | | | Data cannot be extracted. | | | Not reported | | | Not explicit | | | Not reported | | | | | Not reported | | | |
| Njunge (2017) | | ABM was defined as children who had a positive bacterial culture for CSF. Cerebral malaria was defined as children who had peripheral asexual malarial parasites >2500 parasites/μl on blood film19, negative CSF cultures, CSF leukocyte count <10 cells/μl and no CSF biochemical feature of ABM. ABM was defined without respect to parasitaemia, and therefore 5 children had Plasmodium falciparum coincidental infection. | | | | | | | | | | | 37, 22 | | | 43%, 64% | | | Months 35 (9 – 90), 30.5 (13 – 37) | | | Not explicit | | | Not reported | | | | | Not reported | | | |
| Ou (2013) | | 1) TBM: twenty patients definitively diagnosed with TBM (having the disease no longer than 2 weeks). All patients had typical clinical manifestations of TBM and CSF that tested positive for M. tuberculosis DNA according to acid-fast bacillus smears, cultures, or PCR results. The patients were yet to receive treatment. 2) Cryptococcal meningitis: These patients had typical clinical manifestations, positive smear or culture results, and were yet to receive treatment. | | | | | | | | | | | 20, 20. Subsequently 25, 25 ELISA testing. | | | 35%, 40% | | | Mean 32.6 (18 - 52), 35.8 (18 - 65). | | | Not explicit | | | Not reported | | | | | Not reported | | | |
| Sengupta (2015) | | JE IgM in CSF using IgM Capture ELISA kit developed by the National Institute of Virology (Pune) as per the manufacturer’s instructions. WHO criteria. | | | | | | | | | | | 10 | | | 20% | | | Range 4-45 | | | Not explicit | | | Not reported | | | | | Not reported | | | |
| Tiberti (2015) | | Patients suffering from sleeping sickness were diagnosed based on detection of trypanosomes in blood and/or in lymph. HAT stage was then determined through detection of parasites and counting white blood cells (WBC) in CSF, according to the guidelines of the National Sleeping Sickness Control Programs of the countries of sample collection. For the present study, patients were classiﬁed as stage 1 when no parasite was detected in the CSF and the number of CSF WBC was >5/mL, while stage 2 patients were deﬁned by the presence of trypanosomes in the CSF and/or CSF WBC >5/mL, following WHO guidelines. CSF samples collected before treatment. | | | | | | | | | | | 3, 3. Subsequently, 39, 126 ELISA. | | | 100%; not reported for all patients in verification. | | |  | | | Not explicit | | | Not reported | | | | | Not reported | | | |
| **Study** | | **Control Details** | | | | | | **Number of Controls** | **Controls - % Female** | | **Controls - Median Age** | | | **Controls - Ethnicity** | | | **Controls – Comorbidities.** P*regnancy, epilepsy* | | | **Controls - Medications** | **Other (Non-CSF) Types of Samples** | | | | | | | | | | | | |
|  |  |  | | | | | |  |  |  |  |  |  |  |  |  |  |  |  |  | **serum** | | **plasma** | | | **urine** | | **throat** | | | **saliva** | **other** | |
| Angel (2012) | | Patients who had LP performed for a variety of clinical indications. Fever was present in 11/19 control patients. A variety of other diagnoses (postoperative fever, alcohol withdrawal, pneumonia, pyelonephritis) was made in 6 of the 11 cases. Of the 5 remaining cases presenting with fever and headache, 2 had completely negative Lyme blood and/or CSF diagnostic studies and one had a positive serum IgM immunoblot, negative IgG immunoblot, and negative CSF capture EIA studies and was not felt to have central nervous system Lyme disease. Two were diagnosed with non-meningitic viral syndromes based on their clinical, laboratory, and epidemiologic features and resolution without therapy for neuroborreliosis. | | | | | | 19 | 42% | | Mean 44 (IQR 3-83) | | | Not explicit | | | Not reported | | | Not reported | No | | No | | | No | | No | | | No | No | |
| Asano (2011) | | Febrile seizures were defined as 1) seizures with fever and impaired consciousness lasting less than 24 h without neurological sequelae, 2) usually occurring between 3 months and 5 years of age, and 3) without evidence of intracranial infection or defined cause. References cited. | | | | | | 42 | 29% | | Mean age months group 1 29.8 ± 24.9; group 2 44.1 ± 40.6. | | | Not explicit | | | Not reported | | | Not reported | No | | No | | | No | | No | | | No | No | |
| Bonnet (2018) | | CATT positive but Trypanosomes not detected and WCC <5 | | | | | | 3 | 0% | | Mean age 36.67 (SD 27.15) | | | Not explicit | | | Not reported | | | Not reported | Yes | | No | | | Yes | | No | | | Yes | No | |
| Cordeiro (2015) | | Patients who underwent LP for suspected of meningitis, but have a normal CSF profile, spontaneous relief of symptoms. | | | | | | 6 | 33% | | 1.9 (<1 - 33) | | | North-East vs South-East 17% | | | Not reported | | | Not reported | No | | No | | | No | | No | | | No | No | |
| Fraisier (2014) | | C1 group with non-WNV CNS infection for whom paired serum and CSF was available; C2 group with acute headache (AH group), with CSF sample available; C3 group with idiopathic intracranial hypertension (IIH group), with CSF sample; and C4 group apparently healthy French individuals (‘‘healthy group’’). AH and IIH groups had no neurological disorders, did not take any medication, and were considered to have normal CSF. | | | | | | 13, 6, 5, 21. 6&5 = 11 used in the MS. | 38.5, 33.3, 100 and 47.6. 33.3 and 100 used for the MS. | | 48.5 (20–74), 35.1 (21–56), 35.8 (26–44) and 45.4 (24–62). 35.1 (21–56) and 35.8 (26–44) MS. | | | Caucasian. C1 Greek, C2&3 Dutch and C4 French. Note C2&3 were used for MS. | | | Aside from C1 non-WNV CNS infection, C3 IIH, not reported | | | Not reported | Yes | | No | | | No | | No | | | No | No | |
| Gomez-Baena (2017) | | CSF from children admitted to hospital with symptoms of ABM but who were negative after diagnostic tests. Subsequently hospital controls and healthy controls using gel-based methods. | | | | | | 4, 4. 15, 5 gel-based methods. | Not reported | | Not reported | | | Malawian | | | Not reported | | | Not reported | No | | No | | | No | | No | | | No | No | |
| Mu (2015) | | Healthy controls demographically matched with the subjects, with neither active TB nor any neurological complaints. | | | | | | 12 | Data cannot be extracted. | | Data cannot be extracted. | | | Not explicit | | | Not reported | | | Not reported | No | | No | | | No | | No | | | No | No | |
| Njunge (2017) | | No separate control group, two case groups compared. The authors acknowledge this in the discussion, and provide clear rational. | | | | | | - | - | | - | | | - | | | - | | | - | No | | No | | | No | | No | | | No | No | |
| Ou (2013) | | Healthy volunteers | | | | | | 20 | 35% | | Mean 38.4 (16 - 55) | | | Not explicit | | | Not reported | | | Not reported | No | | No | | | No | | No | | | No | No | |
| Sengupta (2015) | | Non-JE AES | | | | | | 10 | 30% | | Range 4.5–50 | | | Not explicit | | | Not reported | | | Not reported | No | | No | | | No | | No | | | No | No | |
| Tiberti (2015) | | Patients at the University Clinics of Kinshasa (D.R.C.) hospitalized for health problems other than HAT and needing a LP as a required procedure for their care. | | | | | | 20 | 70% | | 35 (18.5-45.3) | | | Not explicit | | | Not reported | | | Not reported | No | | Yes | | | No | | No | | | No | No | |
| **Study** | | **Sample Collection, Transport and Storage Details** | | | | | **Sample Preparation** | | | | | | | | | | | | | | | | | | | | | | | | | | |
| Angel (2012) | | Centrifuged, supernatant stored at −70 °C prior to shipping overnight on dry ice and stored at −80 °C. | | | | | CSF proteins denatured with 8 M urea and disulfide bonds were reduced with 5 mM dithiothreitol (DTT) at 37°C for 60 min. Proteins digested with porcine Trypsin overnight at 37 °C. Tryptic peptides cleaned with a 1 mL SPE C18 column. Final peptide concentrations were determined by BCA assay. All tryptic digests were snap frozen in liquid nitrogen and stored at −80 °C until further processing and analysis. | | | | | | | | | | | | | | | | | | | | | | | | | | |
| Asano (2011) | | Centrifuged at 1500 rpm for 5 min removing cells, and frozen on dry ice and stored at -80°C. | | | | | The Protein Chip array condition that produced optimal results was 50 mM Tris-HCl, pH 8.0 binding and wash steps, and this was therefore used in this study. 1.5 ug protein was used. For the analysis of the 4.8 kDa peak, purified using anion ion exchange chromatography and reverse-phase high-performance liquid chromatography. | | | | | | | | | | | | | | | | | | | | | | | | | | |
| Bonnet (2018) | | Stored in liquid nitrogen, transported in dry tanks and further stored at −80 °C. | | | | | Depletion of predominant proteins (α1-Acid Glycoprotein, α1-Antitrypsin, α2-Macroglobulin, Albumin, Apolipoprotein A-I, Apolipoprotein A-II, Fibrinogen, Haptoglobin, IgA, IgG, IgM, Transferrin) was performed. Samples containing 10 μg of protein, after depletion and concentration with an amicon filter, were adjusted to 120 μL of the reaction mixture containing 4 M urea, 1.5 M thiourea and 50 mM tris–HCl pH 8.3. Reduced with 10 mM dithiothreitol for 30 min and then alkylated with 55 mM iodoacetamide for 20 min. Alkylated proteins were first digested with 500 ng of endopeptidase lys-C for 3 h at room temperature. Then diluted with 3 volumes of MilliQ-water and treated with 500 ng of trypsin for 16 h at RT. Enzymatic activity was stopped by addition of formic acid to a final concentration of 3%. Samples were stored at −20 °C until use. For saliva, acetone (100%) precipitation at −20 °C was carried out. Saliva and urine were not depleted to avoid loss of proteins. | | | | | | | | | | | | | | | | | | | | | | | | | | |
| Cordeiro (2015) | | CSF samples kept at 4°C for up to 6 hrs then centrifuged for 10 minutes at 5,000 rpm, and supernatants frozen at -20°C for up to a week and then at -80°C. | | | | | Proteins in aliquots of 300 µL of CSF were precipitated with acetone, the pellets were resuspended in 15µL ultrapure water and the volumes were adjusted to 100 µL with PBS buffer. These 3X concentrated protein solutions were applied to the columns provided with the Albumin & IgG Depletion SpinTrap kit to deplete overabundant proteins. The proteins in the whole volumes eluted from the columns (300 µL) were precipitated again with acetone and solubilized in 50 µL of IEF rehydration buffer [8 M Urea, 2 M Thiourea, 4% CHAPS, 0.0025%]. Protein concentrations were assessed using the Bradford assay. Protein degradation was monitored in silver stained 1D PAGE gels before and after albumin and IgG depletion. | | | | | | | | | | | | | | | | | | | | | | | | | | |
| Fraisier (2014) | | Not reported | | | | | Proteins were precipitated with cold acetone for 2 h at –20uC, centrifuged for 5 min at 16,000g, dissolved in 20 mL of dissolution buffer, denatured, reduced, alkylated and digested with 10 mg of trypsin overnight at 37uC, following manufacturer’s protocol. Peptides labeled with iTRAQ reagents according to manufacturer’s instructions. Before combining the samples, a premix containing an aliquot of each labeled sample, cleaned-up using a ZipTip, was analyzed by MS/MS to check for peptide labeling efficiency with iTRAQ reagents and homogeneity of labeling between each sample. iTRAQ labeled samples were pooled. | | | | | | | | | | | | | | | | | | | | | | | | | | |
| Gomez-Baena (2017) | | Centrifuged within 2 h of collection, supernatant fraction frozen within 4 h of collection, and stored at −80 °C. | | | | | CSF (20 μL) was incubated with RapiGest SF surfactant at a final concentration of 0.05% (w/v) for 10 min at 80 °C in 25 mM ammonium bicarbonate. Samples were then reduced with 3 mM DTT for 10 min at 60 °C, followed by alkylation with 9 mM iodoacetamide for 30 min in the dark at room temperature. Finally, trypsin was added and incubated overnight at 37 °C. To stop the proteolytic reaction and to inactivate and precipitate the detergent, trifluoroacetic acid (final concentration 0.5% (v/v)) was added, followed by incubation for 45 min at 37 °C. Samples were centrifuged at 13,000 g for 15 min and the supernatant peptide fraction analysed. | | | | | | | | | | | | | | | | | | | | | | | | | | |
| Mu (2015) | | Not reported | | | | | Pooled sample (5 ml) desalted and concentrated to 80 µl in a Vivaspin spin column MWCO 5 kDa. 120 mg of protein from each sample dissolved in 30 ml of STD buffer (4% SDS, 100 mM DTT, 150 mM TrisHCl pH 8.0), boiled in water for 5 min, cooled to room temperature, diluted with 200 ml of UA buffer (8 M Urea, 150 mM TrisHCl pH 8.0), and transferred to 30 kDa filter. Samples centrifuged at 14000 g for 15 min and 200 ml UA buffer added. Samples centrifuged again for 15 min at the same conditions. Then, 100 ml of 0.05 M iodoacetamide in UA buffer was added, and the samples were incubated for 20 min in darkness. After 10 min of centrifugation at the above conditions, the filters were washed three times with 100 ml of UA buffer. Then, 100 ml of DS buffer (50 mM triethylammoniumbicarbonate, pH 8.5) was added to the filters, and the samples were centrifuged for 10 min at the above conditions. This step was repeated twice. Finally, 2 mg of trypsin in 40 ml of DS buffer was added to each filter, and the samples were incubated overnight at 37˚C. The resulting peptides were collected by centrifugation. The filters were rinsed with 40 ml of 10 DS buffer again. iTRAQ labeling was performed according to the manufacturer's instructions. The process was repeated twice. | | | | | | | | | | | | | | | | | | | | | | | | | | |
| Njunge (2017) | | Not reported | | | | | 10 μl of CSF denatured in 50 mM ammonium bicarbonate containing 8 M urea. Reduced with 20 mM dithiothreitol at room temperature shaking for 1 hr and alkylated in the dark for 1 h with 65 mM iodoacetamide. Excess iodoacetamide was quenched using 65 mM dithiothreitol. Urea present in the sample was dialyzed out with 50mM ammonium bicarbonate, using 3 kDa amicon filters. Proteins were digested with trypsin overnight (16 hrs) and peptides obtained were desalted using C18 Spin columns, according to manufacturer’s instructions, dried in a Speedvac concentrator, and resuspended in 50 μl loading solvent (97.05% H2O, 2% acetonitrile, 0.05% formic acid). | | | | | | | | | | | | | | | | | | | | | | | | | | |
| Ou (2013) | | Not reported | | | | | 100 μg each of three samples were labelled. iTRAQ labelling was performed in accordance with a kit protocol. After trypsin digestion, iTRAQ reagents in 70 μL ethanol were added separately to each tube and incubated at room temperature for 1 h. All labelled peptide solutions were pooled in a new vial and dried using a rotary vacuum concentrator. Pooled iTRAQ-labelled peptide samples were desalted prior to SCX chromatographic fractionation and LC-MS/MS analysis. | | | | | | | | | | | | | | | | | | | | | | | | | | |
| Sengupta (2015) | | LP within 3 hrs of admission, CSF volume collected was 2–3 ml. | | | | | The pooled CSF samples were concentrate to achieve desire protein concentration and were subjected to high abundant protein depletion using BioRad ProteoMiner. The enriched proteins were subjected to 2D gel electrophoresis. Visually spotted protein bands were digested using in-gel trypsin digestion protocol. | | | | | | | | | | | | | | | | | | | | | | | | | | |
| Tiberti (2015) | | Cited reference Tiberti et al 2010 - Samples were aliquoted and stored at −80°C or in liquid nitrogen. | | | | | 60 mL CSF per patient was reduced with 50 mM TCEP, alkylated with 400 mM iodoacetamide and digested overnight at 37’C with trypsin after having spiked 0.5 mg per sample of bovine beta-lactoglobulin as an internal control. | | | | | | | | | | | | | | | | | | | | | | | | | | |
| **Study** | | **Depletion** | | **Label** | **Separation** | | | | | **MS machine** | | | | | **MS Method** | | | | | | | | | | | | | | | | | | |
| Angel (2012) | | Yes | | No | 1^st^ dimension fractionation was performed using a SCX column.  2^nd^ dimension separation of fractions using online low pH reversed phase. | | | | | LTQ | | | | | MS was operated in the data-dependent mode with full scan MS spectra (m/z 400–2000) acquired in the LTQ-Orbitrap Velos with resolution of 60,000 at m/z 400 (accumulation target: 1,000,000). | | | | | | | | | | | | | | | | | | |
| Asano (2011) | | No | | No | 2D gels and LC prior to SELDI/ MALDI). | | | | | SELDI-TOF and MALDI-QSTAR | | | | | The ionized proteins were detected and their molecular mass/charge (m/z) ratios determined using time-of-flight (TOF-MS) analysis with a detection range m/z of 1,570-12,230 for low molecular range, 5,733 to 43,240 for mid range and 16,951-147,300 for high molecular range. peptide Mass Finger (PMF) was confirmed using MALDI-TOF MS after trypsin digestion of the 4.8 kDa protein. | | | | | | | | | | | | | | | | | | |
| Bonnet (2018) | | Yes | | No | Online separation using Acclaim PepMap100 C18 pre-column and Pepmap-RSLC Proxeon C18 column. | | | | | Q Exactive Orbitrap | | | | | Full ion scan mode, at a resolution of 70,000 (at m/z 200), with a mass range of m/z 375–1500 and an AGC target of 3 × 106. Fragments were obtained by HCD activation with a collisional energy of 30%, and a quadrupole isolation window of 1.4 Da. MS/MS data were acquired in the Orbitrap cell in a top 20 mode, at a resolution of 17,500, with an AGC target of 2 × 105, with a dynamic exclusion of 30 s. The most intense precursor ions were acquired first by MS/MS. Peptides with unassigned charge states or monocharged were excluded from the MS/MS acquisition. Maximum ion accumulation time was set to 50 ms for MS and 45 ms for MS/MS acquisition. | | | | | | | | | | | | | | | | | | |
| Cordeiro (2015) | | Yes | | No | 2D gels. | | | | | MALDI-ToF | | | | | The MS and MS/MS data were acquired using a laser with an iteration rate of 1 kHz, number of shots used was 2000 for both MS and MS/MS. The 12 most intense peaks whose S/N ratio was more than 2 were selected for MS/MS fragmentation. | | | | | | | | | | | | | | | | | | |
| Fraisier (2014) | | No | | Yes | 1^st^ dimension fractionation was performed using an off-gel system (Agilent 3100 OFFGEL fractionator) into 12 fractions.  2^nd^ dimension online nano-LC (UltiMate 3000 Rapid Separation LC systems). | | | | | LTQ | | | | | LTQ settings used were: voltage 1.4 kV, Capillary temp 275 oC, Acquisition mode; DDA top 10 mode. Resolution: 30K, AGC target: 106, max IT: 500 ms, Collision energy; 35%, MS2-setting: activation time: 10ms, AGC target: 104, max IT: 100ms, isolation window 2Da, exclusion range: 30s. | | | | | | | | | | | | | | | | | | |
| Gomez-Baena (2017) | | No | | No | No fractionation of the in-solution trypstic digested peptdes were performed.  Peptides were separated on a nano-LC with a online peptide trap (C18). | | | | | LTQ-Orbitrap Velos | | | | | Data were acquired in DDA top 20 mode with 35% normalised collision energy. The dynamic exclusion was 20 sec. | | | | | | | | | | | | | | | | | | |
| Mu (2015) | | No | | Yes | 1^st^ dimension fractionation was performed using an SCX column.  2^nd^ dimension separation of each fration on a nano-LC low pH. | | | | | Triple-ToF | | | | | Data was aquired in Information dependent acquisition mode, resolution 30 K, mz- 350-1250, dynamic exclusion was 18 s. Inhance iTRAQ function was enabled to calculate CID energy for labelled peptides. | | | | | | | | | | | | | | | | | | |
| Njunge (2017) | | No | | No | The digested peptides were separated on a nano-LC Reversed phase low pH chromatography. | | | | | Q Exactive Orbitrap | | | | | Resolution 70K, AGC target 3e6, M/Z range 400-1800, MS2-settings: resolution 17.5K, AGC target 5e4, isolation window: 1.6 m/z, dynamic exclusion: 30 sec. | | | | | | | | | | | | | | | | | | |
| Ou (2013) | | No | | Yes | 1^st^ dimension fractionation was performed using an SCX column.  2^nd^ dimension separation of each fration on a nano-LC low pH. | | | | | QSTAR | | | | | The data were acquired using a survey sacn in to mode with a scan range m/z 400-1800 and MS2 scan arnge: m/z 100-2000. | | | | | | | | | | | | | | | | | | |
| Sengupta (2015) | | No | | No | 1^st^ dimension isoelectric focusing was performed using immobilized pH gradient.  2^nd^ dimension 2D gel. JE specific protein spots were excised. | | | | | MALDI-ToF | | | | | MS settings not described | | | | | | | | | | | | | | | | | | |
| Tiberti (2015) | | No | | Yes | 1^st^ dimension fractionation was performed using an off-gel system (Agilent 3100 OFFGEL fractionator) into 12 fractions.  2^nd^ dimension online nano-LC low pH RP | | | | | LTQ Orbitrap Velos | | | | | Data were acquied using a survey scan. MS1: resolution 60K, mz/ scan range: 400-2000, Top 3 mS/MS, resolution: 7.5 Kmax IT: 750 ms, CID -35% and HCD-60%. | | | | | | | | | | | | | | | | | | |
| **Study** | | **Data Analysis** | | | | | | | | | | | | | | | | | | | | | | | | | **Data Uploaded**  *e.g. PRIDE* | | | | | | |
| Angel (2012) | | Identification with SEQUEST (v27 revision 12), quantification & statistical analysis with in-house DAnTE. | | | | | | | | | | | | | | | | | | | | | | | | | Not reported | | | | | | |
| Asano (2011) | | Identification with Mascot, quantification and statistical analysis with Protein Chip Data Manager software | | | | | | | | | | | | | | | | | | | | | | | | | Not reported | | | | | | |
| Bonnet (2018) | | Identification with Proteome Discoverer software (Thermoscientific,version2.1) and with Mascot search engine (Matrix Science, version5.1). Progenesis QI for Proteomics software (version 4.0, Waters) was used for the relative quantification of the protein abundances by using co-detection to eliminate missing values. | | | | | | | | | | | | | | | | | | | | | | | | | PRIDE (PXD007842) | | | | | | |
| Cordeiro (2015) | | Raw data were converted into a single *.mgf format peaklist file by Proteome Discoverer 1.1 (Thermo Fisher Scientific, Waltham, MA) using default parameters. Independent *.mgf files for each sample were searched with MASCOT search engine (version 2.5.1, Matrix Science), using trypsin as specific enzyme, carbamidomethylation of cysteine as fixed modification, methionine oxidation as variable modification and one trypsin missed cleavage, a mass tolerance of 10 ppm for precursors and 0.6 Da for fragment ions. The false discovery rate (FDR) was calculated using the decoy database tool in MASCOT. Only those proteins identified by at least 2 significant peptides, and at a FDR <1% were accepted. The minimum list of proteins explaining the set of peptides identified was built using the Report builder in MASCOT. Proteins were quantified using Progenesis QI software v2.0. Quantification was based on unique peptides, raw abundances and non-conflicting features. The abundance of a peptide was calculated from the peak area and the protein abundance was calculated from the sum of all unique peptide abundances for a specific protein across each sample. Features with positive charge states between 2 and 5, and three or more isotopic peaks were taken to further analysis. Different biological samples were grouped as control or infected. A merged peaklist generated by Progenesis QI was searched against the database described in the section above, using MASCOT search engine (version 2.5.1, Matrix Science) and the same search parameters. A cut off score of 20 was applied after manually evaluating the quality of the lowest scored peptides. Proteins containing similar peptides were grouped into families. The criteria to consider a protein to be significantly up- or down-regulated were: a fold change between groups greater than a 2-fold using at least 2 unique peptides, p value < 0.05 and q value < 0.05, calculated in Progenesis QI. | | | | | | | | | | | | | | | | | | | | | | | | | Not reported | | | | | | |
| Fraisier (2014) | | Raw files generated from MS analysis were combined and processed with Proteome Discoverer v 1.3 (Thermo Fisher Scientific, Waltham, MA, USA). Protein identification and quantification were carried out using the Mascot search engine (v.2.3; Matrix Science, Boston, MA, USA) and SEQUEST (v.28.0.0.0; University of Washington) through Proteome Discoverer v 1.3 (Thermo Scientific). The search was performed against the Homo Sapiens Sapiens database containing 20257 sequences (from SwissProt, May 24rd, 2012). | | | | | | | | | | | | | | | | | | | | | | | | | Not reported | | | | | | |
| Gomez-Baena (2017) | | Raw data were converted into a single *.mgf format peaklist file by Proteome Discoverer 1.1 (Thermo Fisher Scientific, Waltham, MA) using default parameters. Independent *.mgf files for each sample were searched against a database composed of reviewed entries of Human Uniprot database (version 20151209; 20,187 entries) and Streptococcus pneumoniae reference strain ATCC BAA-255/R6 (version 20151209; 2,030 entries) with MASCOT search engine (version 2.5.1, Matrix Science), using trypsin as specific enzyme, carbamidomethylation of cysteine as fixed modification, methionine oxidation as variable modification and one trypsin missed cleavage, a mass tolerance of 10 ppm for precursors and 0.6 Da for fragment ions. The false discovery rate (FDR) was calculated using the decoy database tool in MASCOT. Only those proteins identified by at least 2 significant peptides, and at a FDR <1% were accepted. | | | | | | | | | | | | | | | | | | | | | | | | | ProteomeXchange via PRIDE (PXD004219) | | | | | | |
| Mu (2015) | | The data were processed using Protein Pilot (version 3.0). The qunatitation was based on the ratios of the signature mass tag of iTRAQ obtained from MS/MS fragmentation. The identification was performed with > 95% confidence and the fold change >2 were considered as significantly changed proetins. | | | | | | | | | | | | | | | | | | | | | | | | | Not reported | | | | | | |
| Njunge (2017) | | Raw data were analysed by MaxQuant software version 1.5.3.3020 by searching against the human Uniprot FASTA database (downloaded February 2014) using the Andromeda search engine. Cysteine carbamidomethylation was set as a fixed modification and N-terminal acetylation and methionine oxidations as variable modifications. The false discovery rate (FDR) was set to 0.01 for both proteins and peptides with a minimum length of seven amino acids and was determined by searching a decoy database. Enzyme specificity was set as C-terminal to arginine and lysine with trypsin as the protease. A maximum of two missed cleavages were allowed in the database search. Peptide identification was performed with an allowed initial precursor mass deviation of up to 7 ppm and an allowed fragment mass deviation of up to 20 ppm. The label free quantification (LFQ) algorithm in MaxQuant was used to obtain quantification intensity values. | | | | | | | | | | | | | | | | | | | | | | | | | ProteomeXchange (PXD006357) via MassIVE | | | | | | |
| Ou (2013) | | The acquired data were processed with Protein Pilot (version 4.0) using Mascot as search engine against Human taxonomy. | | | | | | | | | | | | | | | | | | | | | | | | | Not reported | | | | | | |
| Sengupta (2015) | | Acquired combined MS and MS/MS spectra were analyzed with ProteinPilot 4.0 Software using MASCOT v 2.3.02 search engine from matrix sciences against the taxonomy Homo sapiens. The peak list was searched against the taxonomy Homo sapiens at protein sequence Database: UniProtKB-SwissProt sprot_2014-04-16 (544996 sequences; 193815432 residues) Search parameters were as follows: Digestion: trypsin with one missed cleavage; Fixed modification: carbamidomethyl (c); variable modification: oxidation (m); peptide mass tolerance: 100ppm for precursor ion and 0.8 Da for fragment ion with +1 charge state; instrument: MALDI-TOF-TOF | | | | | | | | | | | | | | | | | | | | | | | | | Not reported | | | | | | |
| Tiberti (2015) | | Protein identiﬁcation was done using EasyProt platform v2.3. Peak lists were generated from raw data using ReadW software. After peak list generation, the CID and HCD spectra were merged for simultaneous identiﬁcation and quantiﬁcation. Peptide spectral matches were searched against Swiss–Prot/UniProt database (Version 13 June 2012, 5360489 entries), by choosing Homo sapiens taxonomy. | | | | | | | | | | | | | | | | | | | | | | | | | ProteomeXchange via PRIDE (PXD001082) | | | | | | |
| **Study** | | **Biomarkers** | | | | | | | | | | | | | | | | | | | | | | | | | | | | | |  |  |
|  | | **Criteria** | | | **Results** | | | | **Further Details** | | | | | | | | | | | | | | | | | | | | | | |  |  |
| Angel (2012) | | ANOVA (P value <0.05); AUROC >=0.8. | | | 13 host proteins differentially expressed between cases vs. controls: Secreted phosphoprotein 1, EGF-containing fibulin-like extracellular matrix protein 1, Myoglobin, POTE ankyrin domain family member I, Actin, cytoplasmic 2, Lysozyme, Complement C1q C & B, Ig κ variable 3D-15 & 3-20, IgG Fc binding protein, Vitronectin, α-2- macroglobulin. | | | | 247 proteins identified, 108 different in cases vs controls (60 increased, 48 decreased). 13 had favourable AUROC (>0.8). 53 proteins uniquely differentiated Lyme disease, of which 37 previously identified in the plasma proteome. Data extraction from main text, and supplement 5, looking for unique proteins different in the cases and controls, not seen in other diseases or plasma, identifies one protein = ACTG1. | | | | | | | | | | | | | | | | | | | | | | |  |  |
| Asano (2011) | | Kruskal-Wallis H test, Mann-Whitney test with Bonferroni-Dunn correction (P value <0.05). | | | 1 host protein differentially expressed in between cases vs. controls: Peptide fragment from the neurosecretory protein VGF precursor. | | | | 15 peaks identified and differentially expressed (9 upregulated and 6 downregulated). Among these peaks, the peak at m/z 4810 contributed the most to the separation of the two groups. | | | | | | | | | | | | | | | | | | | | | | |  |  |
| Bonnet (2018) | | ANOVA (P value <0.05) | | | 37 host proteins differentially expressed between groups and with a biological role^.^ 3 host proteins selected for verification: Neuroserpin, neogenin and secretogranin 2; only 1 host protein, neuroserpin, detected by ELISA, and this differentiated between early and late stage disease. | | | | 491 proteins identified, 159 different in groups, 37 fulfilled criteria. | | | | | | | | | | | | | | | | | | | | | | |  |  |
| Cordeiro (2015) | | Qualitative analysis - proteins present in 11/12 gels per group, and not present in another group | | | 4 host proteins differentiated between groups and used to develop a predictive model of meningitis: Apolipoprotein A-I (present in all causes of meningitis and not controls), C-reactive protein & Complement C3 (present in bacterial meningitis and not viral), Kininogen-1 (present in Meningococcal meningitis). | | | | 695 protein spots subjected to MS, 553 (80%) identified, corresponding to 117 distinct proteins. 20 identified proteins occurred in the intersection subsets of the case groups. Most of the aetiology-specific spots ruled out as potential biomarkers after identification by MS, since the same corresponding protein occurred in other positions of the gels. 4 proteins used in a predictive model. | | | | | | | | | | | | | | | | | | | | | | |  |  |
| Fraisier (2014) | | Kruskal-Wallis and Mann-Whitney U tests, P value <0.05 | | | 47 host proteins differentially expressed in cases vs controls during discovery. 1 host protein, Defensin α-1, selected based on its high fold-change, possible functional association with WNV pathobiology, potential use as severe infection biomarker, and availability of ELISA. | | | | 470 proteins identified, 51 proteins were different in the WNND vs controls, and of these 47 selected. The large majority of these proteins were upregulated (42 and five proteins up- and down-regulated, respectively), and fold-changes were much higher for up- (ranging from 2.0 to 12.9) than for down-regulated proteins (ranging from 22.0 to 22.2). | | | | | | | | | | | | | | | | | | | | | | |  |  |
| Gomez-Baena (2017) | | ANOVA, P value <0.05 | | | 134 host proteins and 6 Streptococcus proteins differentially expressed in cases vs controls during discovery. 5 host proteins selected and confirmed by western blotting: Myeloperoxidase, S100 calcium binding protein A9, Cathelicidin antimicrobial peptide, Ceruloplasmin and Cystatin C. | | | | 214 protein groups differentially expressed in the first cohort and 234 protein groups in the second, using the criteria of a minimum FC>2, p value < 0.05, q value < 0.05 and quantification based on >2= unique peptides. Comparing the cohorts reduced proteins to 134 host (human) and six Streptococcus protein groups that were common to the two cohorts. Proteins selected based on confidence of protein identification, the magnitude of change, extent of protein coverage and putative role in pathology. | | | | | | | | | | | | | | | | | | | | | | |  |  |
| Mu (2015) | | Not reported, P value <0.05. | | | 81 host proteins differentially expressed in cases vs controls.  2 host proteins selected for their role in lipid metabolism and confirmed by western blotting and ELISA | | | | 572 unique proteins identified according to the criteria, 81 differential proteins with a ±1.2 fold-change and p value < 0.05 selected and analysed for overrepresentation of pathways to obtain insights into the functional differences between the CSF proteomes of TBM and HC subjects. | | | | | | | | | | | | | | | | | | | | | | |  |  |
| Njunge (2017) | | Mann Whitney test, P value <0.05, AUROC >0.9 | | | 52 host proteins differentially expressed in the two groups. 2 host proteins identified with sensitivity >98% and specificity = 1: Myeloperoxidase and Lactotransferrin | | | | 708 proteins identified, of which 183 proteins commonly expressed in both ABM and CM. 160 proteins quantified in >50% within each group and selected for further analysis. Of these, 32 not quantified in CM, while two proteins not quantified in ABM. | | | | | | | | | | | | | | | | | | | | | | |  |  |
| Ou (2013) | | Not reported, P value <0.05. | | | 9 host proteins differentially expressed in cases vs controls.  2 host proteins selected for and confirmed by western blotting and ELISA: Apolipoprotein B and S100 calcium binding protein A8 | | | | 208 proteins identified and quantified. ELISA performed on a separate sample. | | | | | | | | | | | | | | | | | | | | | | |  |  |
| Sengupta (2015) | | Qualitative analysis of proteins visualised only in JEV cases | | | 7 host proteins identified only in cases: Serum albumin, Vitamin D-binding protein, Fibrinogen gamma chain, Fibrinogen beta chain, Fibrinogen beta chain, Complement C3 & C4b, Actin cytoplasmic-1. | | | | 16 proteins: predominantly DBP, fibrinogen gamma chain, fibrinogen beta chain, complement C4-B, complement C3 and cytoplasmic actin. Mostly members of the albumin multigene family. The levels of two pro-inflammatory cytokines IL-1β and TNFα significantly elevated. | | | | | | | | | | | | | | | | | | | | | | |  |  |
| Tiberti (2015) | | Kruskal-Wallis H test, Mann-Whitney test with Bonferroni-Dunn correction (P value <0.05). | | | 11 host proteins differentially expressed between groups.  3 proteins were selected for further veriﬁcation, based on their TMT ratios as well as on their appearance in the pathways of interest: C-reactive protein, Orosomucoid-1 and Complement component 9. | | | | 239 CSF human proteins with at least 2 unique peptides and <1% false discovery rate (FDR). The ﬁnal list of quantiﬁed human proteins corresponded to 222 entries. 11 proteins resulted to be signiﬁcantly differentially expressed (8 up and 3 down-regulated) between *rhodesiense* and *gambiense* samples. No parasite protein was identiﬁed, probably as a consequence of CSF centrifugation prior to examination. | | | | | | | | | | | | | | | | | | | | | | |  |  |
| **Study** | | **Performance of Biomarker**  *AUROC if reported* | | | | | | | | | | **Pathway Analysis** | | | | | | **Discussion**  *Results, limitations, interpretation, generalisability* | | | | | | **Further work**  *Publications & personal contact* | | | | | **Funding** | | | |  |
| Angel (2012) | | AUROC reported as 0.8 for 13 proteins, however only one is not seen in other diseases or in plasma. | | | | | | | | | | Reported; Ingenuity Pathways Analysis (IPA) | | | | | | Discussion of the role of proteins, how they might be involved in pathogenesis, and discussion of limitations. | | | | | | None identified | | | | | Reported | | | |  |
| Asano (2011) | | - | | | | | | | | | | Not reported | | | | | | Discussion of the role of proteins, how they might be involved in pathogenesis. No discussion of the limitations. | | | | | | None identified | | | | | Reported | | | |  |
| Bonnet (2018) | | For neogenin and secretogranin 2, the amount was too low to be detected. AUROC reported as 0.72 for neuroserpin. It was determined that a level of CSF neuroserpin below 4.99 ng/mL was indicative of a patient having S2 disease, with sensitivity: 94% and specificity: 58% | | | | | | | | | | Not reported | | | | | | Discussion of the role of proteins, how they might be involved in pathogenesis, and discussion of limitations. | | | | | | None identified | | | | | Reported | | | |  |
| Cordeiro (2015) | | - | | | | | | | | | | Reported; Enrichment Analysis | | | | | | Discussion of the role of proteins, how they might be involved in pathogenesis. No discussion of the limitations. | | | | | | None identified | | | | | Reported | | | |  |
| Fraisier (2014) | | The application of a ROC curve allowed defining the threshold value to distinguish WNND from WNF patients at the serum level (0.668 mg/mL) with sensitivity and specificity of 55.6% and 78.3% with serum. | | | | | | | | | | Reported; IPA | | | | | | Discussion of the role of proteins, how they might be involved in pathogenesis. No discussion of the limitations. | | | | | | None identified | | | | | Reported | | | |  |
| Gomez-Baena (2017) | | - | | | | | | | | | | Reported; Gorilla | | | | | | Discussion of the role of proteins, how they might be involved in pathogenesis. No discussion of the limitations. | | | | | | None identified | | | | | Reported | | | |  |
| Mu (2015) | | AUROC 0.906, with sensitivity 89% and specificity 92%. | | | | | | | | | | Reported; DAVID Bioinformatics Resources v6.7, GO, Kyoto Encyclopaedia of Genes & Genomes | | | | | | Discussion of the role of proteins, how they might be involved in pathogenesis. No discussion of the limitations. | | | | | | None identified | | | | | Reported | | | |  |
| Njunge (2017) | | AUROC 0.98, 1, sensitivity 100%, 97% and specificity 100%, 100% for Lactotransferrin and Myeloperoxidase respectively. | | | | | | | | | | Not reported | | | | | | Discussion of the role of proteins, how they might be involved in pathogenesis, and discussion of limitations. | | | | | | None identified | | | | | Reported | | | |  |
| Ou (2013) | | - | | | | | | | | | | Reported; GO | | | | | | Discussion of the role of proteins, how they might be involved in pathogenesis. No discussion of the limitations. | | | | | | None identified | | | | | Reported | | | |  |
| Sengupta (2015) | | - | | | | | | | | | | Reported; STRING v10 | | | | | | Discussion of the role of proteins, how they might be involved in pathogenesis. No discussion of the limitations. | | | | | | None identified | | | | | Reported | | | |  |
| Tiberti (2015) | | - | | | | | | | | | | Reported; GO, IPA | | | | | | Discussion of the role of proteins, how they might be involved in pathogenesis, and discussion of limitations. | | | | | | None identified | | | | | Reported | | | |  |
